# Phylogenomic Analyses Reveal the Evolutionary Origin of the Inhibin α-Subunit, a Unique TGFβ Superfamily Antagonist

**DOI:** 10.1371/journal.pone.0009457

**Published:** 2010-03-04

**Authors:** Jie Zhu, Edward L. Braun, Satomi Kohno, Monica Antenos, Eugene Y. Xu, Robert W. Cook, S. Jack Lin, Brandon C. Moore, Louis J. Guillette, Theodore S. Jardetzky, Teresa K. Woodruff

**Affiliations:** 1 Department of Obstetrics and Gynecology, Feinberg School of Medicine, Northwestern University, Chicago, Illinois, United States of America; 2 Department of Biology, University of Florida, Gainesville, Florida, United States of America; 3 Department of Structural Biology, Stanford University School of Medicine, Stanford, California, United States of America; Cincinnati Children's Research Foundation, United States of America

## Abstract

Transforming growth factor-beta (TGFβ) homologues form a diverse superfamily that arose early in animal evolution and control cellular function through membrane-spanning, conserved serine-threonine kinases (RII and RI receptors). Activin and inhibin are related dimers within the TGFβ superfamily that share a common β-subunit. The evolution of the inhibin α-subunit created the only antagonist within the TGFβ superfamily and the only member known to act as an endocrine hormone. This hormone introduced a new level of complexity and control to vertebrate reproductive function. The novel functions of the inhibin α-subunit appear to reflect specific insertion-deletion changes within the inhibin β-subunit that occurred during evolution. Using phylogenomic analysis, we correlated specific insertions with the acquisition of distinct functions that underlie the phenotypic complexity of vertebrate reproductive processes. This phylogenomic approach presents a new way of understanding the structure-function relationships between inhibin, activin, and the larger TGFβ superfamily.

## Introduction

The common ancestor of the TGFβ superfamily arose early in animal evolution, before bilaterians and cnidarians diverged [1]. The TGFβ gene family is large and has members that encode dimeric ligands of membrane-spanning serine-threonine kinases (RII and RI receptors) that regulate diverse cellular functions at the autocrine or paracrine level [2], [3]. The 42 members of the TGFβ superfamily include the TGFβs, bone morphogenic proteins (BMPs), growth and differentiation factors (GDFs), activins, inhibins, anti-Müllerian hormone (AMH), Nodal, and the leftys [4], [5]. TGFβ superfamily members are synthesized as pre-prohormones that are extensively processed to a ‘mature’ C-terminal active form (Fig. S1). Each processed monomer has six to nine conserved cysteine residues in the mature domain that form a cysteine knot motif, which is characteristic of all TGFβ superfamily members (reviewed in [6]) and is crucial to both the three-dimensional structure of the monomer and formation of the active dimer. Activin and inhibin are members of the TGFβ superfamily linked by structure, function and phylogeny. Inhibin and its unique α-subunit adhere to many but not all of the rules established for the other superfamily ligands. Following the stepwise evolution of the inhibin α-subunit provides a unique vantage point in understanding the constraints on the other members of this extraordinarily powerful and highly conserved family of ligands.

The activin subgroup of the TGFβ superfamily comprises four polypeptide subunits in mammals: β_A_, β_B_, β_C_, and β_E_ [7], [8] are 112–116 amino acids in length and contain nine conserved cysteines. Activins are homo- and heterodimers of these subunits (activin A and activin B are the dominant ligands of the reproductive axis). Activins are made locally and constitutively and then bind cell surface activin type II receptors (ActRIIA/B) and subsequently stimulate activin type I receptors (ActRIA/IB) to initiate intracellular signaling events [3], [9]. Inhibin is the only TGFβ superfamily ligand assembled from two relatively dissimilar subunits; it shares a β-subunit with activin but couples with a dissimilar α-subunit [10]. In mammals, the inhibins (inhibin A [α-β_A_] and inhibin B [α-β_B_]) also bind the activin type II receptors (ActRIIA/B) in addition to an accessory binding protein called betaglycan, but do not activate downstream signaling proteins [11], [12], [13]. In this manner, inhibin acts as an antagonist that blocks the ability of activin to bind and activate its receptors.

The dominant role of activin is to locally and constitutively stimulate follicle-stimulating hormone (FSH) synthesis and secretion from pituitary gonadotrope cells [14], [15]. The pituitary hormone FSH is released in a cyclic manner (monthly in humans and every 4 days in the mouse) and activates a new set of growing ovarian follicles. In direct response to FSH, the follicles produce inhibins that are secreted into the peripheral circulation and block activin-regulated receptors on the gonadotrope. In this manner, peripheral inhibin blocks activin-dependent FSH production at precise times during the reproductive cycle in a classical negative feedback loop [16], [17], [18], [19]. Loss of activin or inhibin is catastrophic to the reproductive cycle of all mammals [20].

The activin β_A−_ and β_B−_subunits show a high degree of sequence conservation in all invertebrates and vertebrates that have been examined [21]. In contrast, the α-subunit of inhibin does not appear in lower invertebrates and mammalian orthologues are quite different from the β-subunits except in the spacing of the cysteines, which has lead to speculation regarding the origin of the α-subunit and the evolution of its functional characteristics. For example, the mature avian α-subunit is 113 amino acids in length (similar to the β-subunits) but contains seven cysteines (the mature β-subunit contains nine cysteines) [22]. Moreover, all β-subunits have a helical ‘wrist region’ necessary for receptor binding [23]. This wrist region is the location of an intriguing difference between α- and β-subunits that is unexpected from an evolutionary perspective. This wrist region is missing from the α-subunits of non-mammalian vertebrates whereas a precise insertion that effectively replaces the wrist region has occurred at this position in mammalian α-subunits [22]. However, the mammalian insertion is a proline-rich sequence that is not homologous to the wrist region helix of β-subunits. Moreover, both avian and mammalian mature α-subunit proteins have an extended N-terminus and antibodies directed at this region neutralize the biological activity of the hormone [24], [25]. These structural domains provide clues to the function of the ligands and a roadmap of evolutionarily flexible domains.

The goal of this phylogenomic analysis of the inhibin/activin genes was to establish the timing of gene duplications, as well as the number and types of changes that occurred after duplication. If the inhibin α-subunit arose from a β-subunit ancestor around the origin of the vertebrates, what were the steps that converted the activin β-subunit into an α-subunit? Sequence changes that occurred after the origin of the inhibin α-subunit should represent changes associated with the acquisition of novel functions of this subunit. Is it possible to account for the unique functions of the inhibin α-subunit not present in the activin β-subunit or any other TGFβ ligand at the sequence level? Specific functions unique to the inhibin α-subunit are the ability to heterodimerize exclusively with activin β-subunits (forming α-β dimers) but not homodimerize (α-α) [26] and to act as an antagonist of activin. Can we assign these functions through an analysis of the structural domains that were iteratively assigned to the α-subunit during animal evolution? These questions can be addressed by phylogenomic analysis and the construction of a set of inhibin ligands based on the results of our phylogenomic analysis to rationally forward and reverse engineer molecules that mimic evolutionary intermediates of the α-subunit. Combining a structure-function analysis with phylogenomics provides a powerful way to understand the contributions of iterative evolutionary changes that contributed to the development of complex reproductive strategies in vertebrates.

## Results

### Regions of Interest and Models of Evolutionary Modification

**Figure 1:**
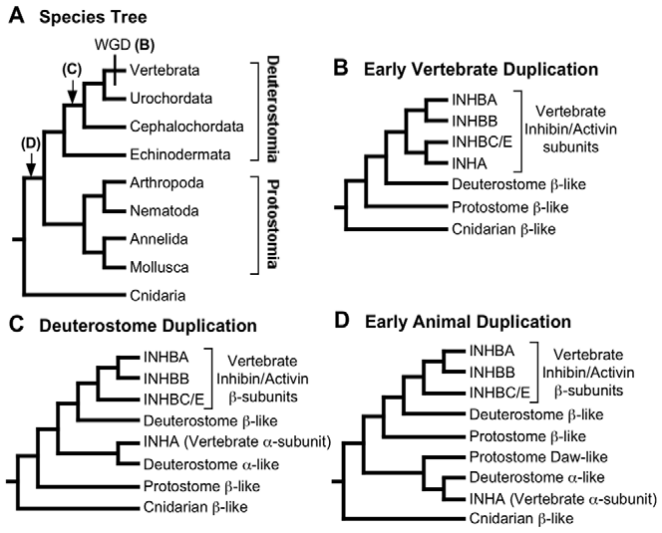
Plausible models of evolution for the inhibin/activin gene family. **A**) Simplified species tree for animals based upon recent large-scale analyses [93], [94], [95]. “WGD” indicates the position of the whole genome duplications [29] uniting vertebrates and letters indicate alternative models for the origin of the α-subunit (the letters used correspond to the parts of this figure). **B**) Cladogram showing the expected inhibin/activin phylogeny given the “early vertebrate duplication” model. Two rounds of WGD have been suggested to characterize vertebrates [28], [29], [30] and we show the only topology consistent both with two rounds of WGD and a clade containing INHBA-INHBB (the latter clade is strongly-supported in this study and in other studies; [27], [96]. **C**) Cladogram showing the expected phylogeny of inhibin/activin genes given the “deuterostome duplication” model, which places the α-subunit origin before the early vertebrate WGDs. Several versions of this model are possible (i.e., the α-subunit origin could predate the divergence of vertebrates from urochordates, cephalochordates, or even echinoderms). **D**) Cladogram showing the expected inhibin/activin phylogeny given the “early animal duplication” model, which places the α-subunit origin before the divergence of deuterosomes and protostomes. The “Dawdle orthologue” hypothesis is a version of this model. Additional duplications may have occurred in any of these models. Biased estimation of the gene tree or sampling variance may cause estimates of the gene tree to deviate from any of these idealized model trees.

A number of plausible hypotheses for the evolution of vertebrate inhibin/activin genes are possible (Fig. 1), most of which postulate the origin of the critical α-subunit occurred after duplication of a gene encoding β-subunit-like TGFβ homolog. Indeed, the genomic location of the human INHA gene (chromosome 2q), which encodes the inhibin α-subunit, suggests a specific version of this “β-subunit duplication” model since INHA is located in a region with extensive intragenomic homology to chromosomes 7, 12q, and 17q centered on paralogous Hox gene clusters [27]. The origin of these paralogous Hox clusters has been suggested to reflect two whole genome duplications (WGDs) early in vertebrate evolution [28], [29], [30] (Fig. 1A). Human β-subunit genes are located near three of these Hox clusters on chromosomes 2q (INHBB, encoding β_B_), 7p (INHBA, encoding β_A_), and 12q (the adjacent INHBC and INHBE genes, encoding β_C_ and β_E_) (Fig. 1B). This “early vertebrate β-subunit duplication” model of inhibin/activin evolution suggests neo-functionalization (the origin of a novel function; [31] immediately after duplication. The hypothesis that the vertebrate genome arose by WGD is not universally accepted [32], [33], although a variant of the model of inhibin/activin evolution involving the duplication of genomic blocks rather than WGD remains possible. Alternatives to the early vertebrate β-subunit duplication model, involving either an earlier origin within the deuterostomes (Fig. 1C) or even an origin before the divergence of protostomes and deuterostomes (Fig. 1D), are also possible. In fact, one such alternative is suggested by the existence of a clade of α-subunit and insect Dawdle homologs (also called “activin-like proteins”) in a phylogenetic analysis of TGFβ homologues [34]. This “Dawdle orthologue” hypothesis can be viewed as a specific version of the “early animal β-subunit duplication” model (Fig. 1D). Either of the alternative models suggests a relatively early duplication that established β-subunit paralogues that subsequently underwent changes and acquired specific functions to become an α-subunit. The major open question regarding the evolution of the vertebrate inhibin/activin gene family are when the duplication that led to the α-subunit occurred and what the implications of that timing were for functional changes that led to the origin of inhibin.

### Phylogenomics Reveals the Origin of the Inhibin α-Subunit

**Figure 2:**
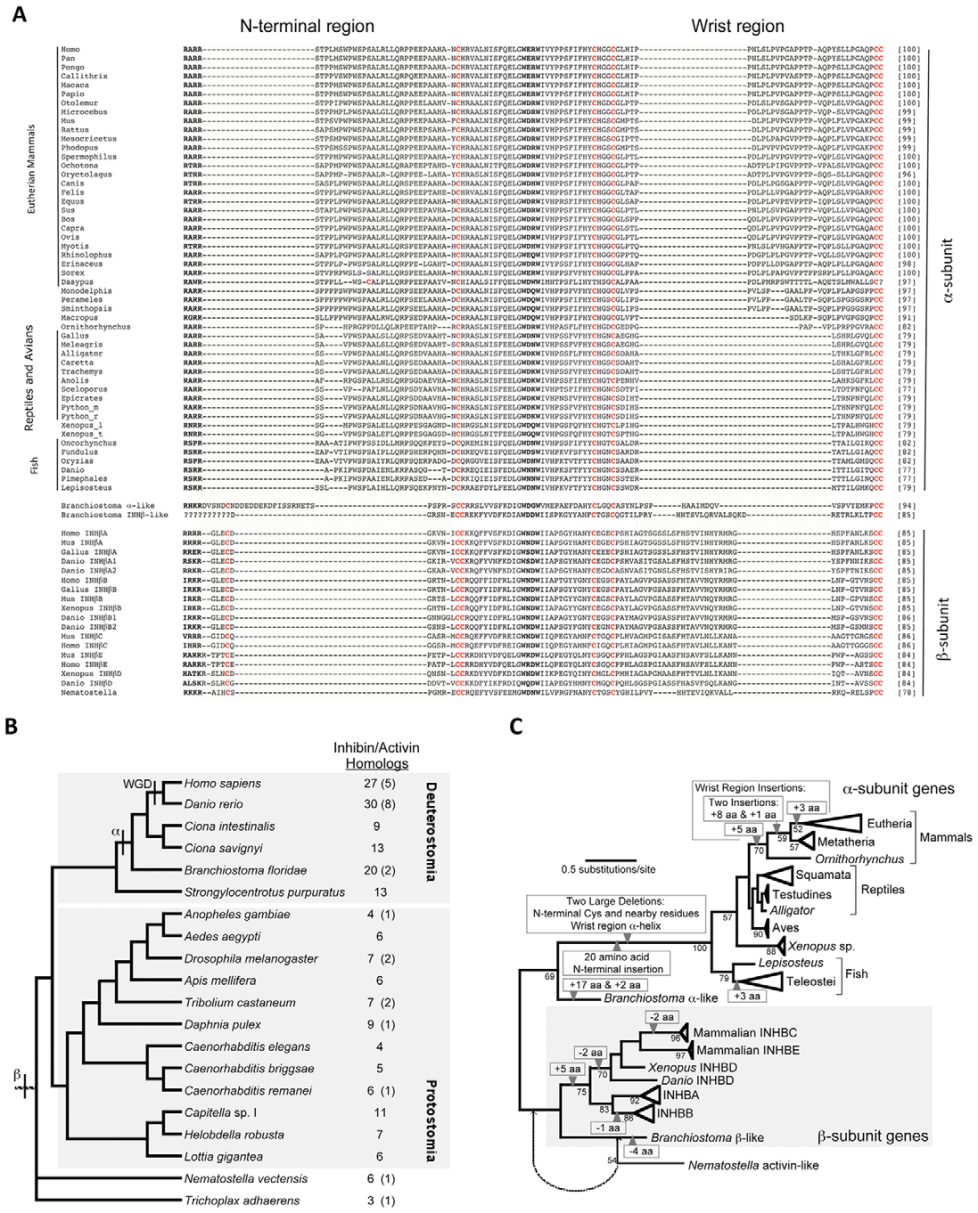
Phylogenomic analyses revealed a complex series of indels that correlated with major events in the evolution of the inhibin α-subunit. **A**) Alignment of mature inhibin/activin proteins showing expanded sampling within the vertebrates for the α-subunit. The alignments were optimized based upon the highly conserved cysteine residues [97] and the highly conserved W-X-X-W motif and R-X-X-R proconvertase enzyme cleavage site (bold). N-terminal region and wrist region were highlighted with grey shade. **B**) Cladogram showing evolutionary relationships among animals with annotated genome sequences available using a topology based upon a consensus of recent analyses [93], [94], [95]. The two major clades of bilaterian animals (deuterostomes and protostomes) are highlighted. Numbers of proteins that exhibit clear homology to inhibin/activin queries in BLASTP searches are shown to the right, with the number of those proteins that have a human inhibin/activin α- or β-subunit as their top hit when used as a query in BLASTP searches of human proteins indicated in parentheses. Thus, numbers in parentheses reflect the number of proteins that are candidates for inhibin/activin α- or β-subunits using a bidirectional BLAST criterion. The likely origins of α- and β-subunits based upon the phylogenomic analyses reported here are indicated using the relevant Greek letters, and the timing of the whole genome duplications uniting vertebrates are indicated as “WGD”. The branch at the base of the tree is hatched because the position of the root is unclear [93], [94], [95]. **C**) Schematic of the ML estimates the phylogeny for inhibin/activin proteins, emphasizing the occurrence of indels in the mature protein region during the evolution of the gene family. Support for specific groups is indicated as the percentage of 100 bootstrap replicates, with only values ≥50% indicated. The starlet sea anemone (*Nematostella*) activin homolog is the sister of the lancelet (*Branchiostoma*) β-like protein and is indicated using a light line since it is probably placed incorrectly (note that bootstrap support is limited). The dashed arrow indicates the likely position of the sea anemone activin; this position is more likely because it minimizes the number of gene duplications and losses given the likely organismal phylogeny shown in **B**). A detailed version of this phylogeny is provided in **Fig. S3**.

To distinguish among alternative models for the origin of the inhibin α-subunit, we identified genes encoding inhibin/activin homologues in annotated genome sequences and aligned the TGFβ domains encoded by those genes using conserved structural elements, like the conserved cysteine residues and the W-X-X-W element, as landmarks (data not shown). The large number TGFβ homologues and conflict among previous estimates of TGFβ phylogeny homologues [34], [35], [36] suggest the evolution of the TGFβ superfamily has been complex. To better understand the evolution of this family, we extracted a large set of TGFβ homologues from available animal genome sequences and identified those that had a human inhibin/activin gene as a top hit (this corresponds to the bidirectional BLAST best hit criterion) (Fig. S2A, Table S1). These database searches revealed a number of potential inhibin/activin genes in several different invertebrate groups (Figs. 2A and 2B). However, all putative inhibin/activin genes appeared to have the structural features typical of β-subunits and genes that clearly encode α-subunits could only be detected in vertebrates using BLAST (Figs. 2B and S2A). A phylogenomic analysis using the maximum-likelihood [37] criterion suggested that one of the lancelet (*Branchiostoma floridae*) inhibin/activin genes is likely to represent an α-subunit orthologue (Figs. 2A, 2B, S2B and S2C), a hypothesis further corroborated by the presence of a specific insertion (Fig. S2B). Insertion and deletion (indel) events are typically considered very reliable evolutionary markers [38], so this insertion provides strong evidence for a specific relationship between the lancelet protein and vertebrate α-subunits. Unlike vertebrate α-subunits, which have seven cysteine residues, the putative lancelet α-subunit orthologue has nine cysteines, suggesting it has retained an ancestral condition of the inhibin/activin family that is typical of β-subunits (Fig. S2B). The lancelet α-subunit does have an N-terminal extension, although it is rich in charged residues, unlike the proline-rich N-terminal extension of vertebrate α-subunits (Figs. 2A and S2C). The lancelet α-subunit also has a partial deletion of the wrist helix (Fig. 2A). Since we were unable to identify inhibin/activin genes in other deuterostome invertebrates, like tunicates or sea urchins (Fig. 2B), there must have been multiple instances of gene loss. This is not unexpected since gene loss has played a major role in eukaryotic evolution [39] and one of the lineages highlighted by this study (tunicates) is known to have undergone substantial gene loss [40]. Indeed, the distribution of genes encoding both α- and β-subunits (Fig. 2B) suggests the evolution of the TGFβ superfamily reflects a complex birth-death process with many instances of gene duplication and gene loss.

Our phylogeny is consistent with an origin of the inhibin α-subunit prior to the divergence of vertebrates and cephalochordates (Fig. 2B), and the duplication leading to the vertebrate α- and β-subunit families could be even more ancient (Figs. 1C and 1D). As expected based upon the short sequences being used [41], phylogenetic analyses of the TGFβ superfamily have limited power and we were unable to exclude alternative models with a more ancient origin of the α-subunit lineage (Fig. S2A). Regardless, the duplication that led to the α-subunit is likely to predate the putative WGDs (Fig. 1A) that resulted in the duplications of the regions surrounding the Hox gene clusters, thought to have occurred close to the origin of vertebrates [42], [43]. Since the only clear invertebrate α-subunit is the lancelet sequence, the “deuterostome β-subunit duplication” model (Fig. 1C), which postulates an origin of the α-subunit by duplication of a β-subunit gene within deuterostomes, probably represents the simplest hypothesis for the origin of the gene encoding the inhibin α-subunit.

### The Inhibin α-Subunit Accumulated Major Insertion and Deletion Changes after Duplication

Comparison of α-subunit and β-subunit sequences revealed a surprisingly limited set of sites where the sequences overlap in a clear manner, with many regions aligning very poorly (Figs. 2A and S2D). The deletion event near the critically important wrist region and the complex indels near the extended N-terminal region are of interest from both evolutionary and functional perspectives (Figs. S2C and S2D). Here, we compared the inhibin α- and β_A_-subunits to determine what evolutionary changes in the α-subunit resulted in the substantial expansion of functions among the inhibins and activins within the TGFβ superfamily [6]. Proline-rich regions near the N-terminal region of the mature α-subunit and the wrist region were particularly striking, especially when we examined a large number of inhibin α-subunit sequences from non-mammalian vertebrates to clarify the evolution of these regions (Figs. 2A and S2D, Table S2). The phylogenomic distribution of these proline-rich regions suggests that the N-terminal extension arose relatively early in the evolution of the α-subunit whereas the wrist region arose much later (Figs. 2C and S3). This alignment suggests that indel changes represent a major type of change that differentiate the inhibin α-subunit and β-subunits, and the distribution of the indels suggest they could have played an important role in changing α-subunit function.

The evolutionary histories of the α-subunit and β-subunits differ fundamentally, since there are four mammalian genes encoding β-subunits (INHBA, INHBB, INHBC and INHBE) and a single gene encoding the α-subunit (INHA). The ML estimate of phylogeny for the mature inhibin/activin protein is consistent with an origin of three major β-subunit clades (corresponding to the mammalian β_A_, β_B_, and β_C_/β_E_ groups) early in vertebrate evolution (Fig. 1B), probably during the WGD [29] (Fig. 1A). The β_C_ and β_E_ groups reflect a subsequent gene duplication event that occurred after mammals' divergence from other vertebrate groups (Fig. S2A). In sharp contrast, we identified a single α-subunit in all taxa we examined (Fig. S2) and the phylogeny included many expected groups (major clades like mammals and teleosts were present; Figs. 2C and S3). Although there were areas of incongruence with the accepted vertebrate phylogeny (e.g., the deepest-branching amniotes were birds and a squamate-mammal clade was found) bootstrap support for these apparent conflicts were limited (Figs. 2C and S3). There are two potential explanations for conflict between a gene tree and the expected species tree. First, the conflict could be genuine and reflect processes that lead to incongruence between gene trees and species trees [44]. Second, the conflict could be only apparent, reflecting an inaccurate estimate of the gene tree either due to bias or limited power. We expected phylogenomic analyses of the inhibin/activin family to have limited power given the limited alignment length [41], and we could not reject the likely species tree based upon bootstrap support (Figs. 2C and S3). These results suggest there have been multiple gene duplications during the evolution of the β-subunit but few duplications and losses during the evolution of the α-subunit.

### Mechanisms of α-Subunit Evolution

The most striking differences between vertebrate α- and β-subunits are the loss of two cysteine residues and the presence of several large indels (Figs. 2A and 2C). These changes occurred at distinct times in the evolutionary history of the α-subunit. The lancelet α-subunit, the only likely inhibin α-subunit we could identify in an invertebrate, does have an N-terminal extension despite having nine cysteine residues similar to the β-subunit. However, it is unclear whether the lancelet N-terminal extension is homologous to the vertebrate N-terminal extension, since the lancelet sequence is rich in charged (especially acidic) residues whereas the vertebrate insertion is proline-rich (Fig. S2C). The second major insertion in the α-subunit, which corresponds to amino acids 68–85 of the human protein (Figs. 2A and 2C), is called the wrist region because it falls between the cysteines where the wrist helix in the inhibin β-subunit is located [45], [46]. Non-mammalian inhibin α-subunits (i.e., those from fish, amphibians, reptiles, and birds) lack the wrist region insertion but some type of wrist region insertion is present in all known mammalian inhibin α-subunits (Figs. 2A, 2C and S2D).

Multi-residue insertions like those observed in the α-subunit are thought to be rare genomic changes [38]. Indeed, both of the major indel events can be mapped as single events on the inhibin α-subunit phylogeny (Figs. 2C and S3) and the fact that marsupials and placental mammals share the long wrist region insertion provides further support for the now well-accepted therian clade [47], [48] that excludes prototherians (*contra* initial mitogenomic analyses, which supported a marsupial-prototherian clade; [49]. However, the evolution of the proline-rich wrist region (PWR) appears to have been highly dynamic. There is a relatively short (5 amino acids) insertion in the platypus but longer (8–19 amino acids) insertions are present in therians. The shortest therian insertion (8 amino acids in the Tammar wallaby, *Macropus eugeni*) probably reflects a secondary deletion given the existence of a longer (14 amino acid) insertion in other marsupials and the phylogenomic position of the wallaby (Figs. 2A and 2C). Emphasizing the dynamic nature of this region, the monotreme and therian wrist-region insertions could have been independent or the shorter platypus insertion could reflect a deletion. However, a stepwise model involving multiple insertions is both simple and consistent with the data. The N-terminal insertion is even more complex, since distinctive insertions are present in the lancelet and in vertebrates suggesting multiple origins of an insertion in this region (Figs. 2A, 2C and S2C). Like the wrist region insertion, there appears to have been a number of indels in this region. This includes independent insertions in teleosts and mammals as well as deletions in the proline-rich N-terminal insertion (Fig. 2A). Examination of the inhibin/activin alignment in an explicit phylogenomic framework emphasizes that there have been multiple indels concentrated in two specific regions and that some of the largest insertions in these regions are correlated with specific events (i.e., the α-subunit origin at the base of the vertebrates and the origin of therian mammals (Figs. 2C and S3).

The existence of multiple indels in specific regions of proteins raises questions about their role in the evolution of α-subunit function and the basis for their recurrence in the same regions. Although multi-residue indels are viewed as rare genomic changes that can be especially valuable for defining clades in phylogenomic trees, multiple insertions can occur in the same position in proteins and these insertions are evident even in studies that have used insertions as phylogenetic markers [50], [51]. Protein structural features are likely to constrain insertions to specific parts of proteins, creating the appearance of recurrent indels. Likewise, insertions are likely to share certain features that may make convergent insertions difficult to identify. These common features might include large numbers of hydrophilic residues (because insertions tend to form exposed loops) or residues that can form flexible linkers like proline and glycine. Indeed, the wrist region insertion is both proline-rich and less conserved than the remainder of the mature α-subunit as one might expect if the function of the wrist region is to act as a flexible linker (flexible sequences often evolve at high rates [52]). The N-terminal insertion is more conserved than the wrist region insertion, but it is also proline-rich. Mechanisms of indel mutations remain poorly understood, although a positive correlation between GC-content and coding region indel rate has been documented [53]. Mammalian INHA third codon positions are GC-rich, as are the two major insertions. Indeed, amino acids over-represented in flexible regions (i.e., glycine and proline) have GC-rich codons, suggesting that insertions in GC-rich coding regions could have a higher likelihood of producing a flexible linker that can be tolerated by proteins. Indeed, it is reasonable to postulate a feedback loop in which an insertion creates a flexible linker and a region prone to further indel changes. Regardless of larger-scale differences among genes in indel probability, the base composition of major INHA insertions is consistent with the composition of the gene (i.e., the wrist-region insertions are 93% GC [14 of 15 nt, including 5 of 5 third positions] in the platypus and 75% GC [36 of 48 nt, including 10 of 16 third positions] in the human). Like any novel mutation, indels could be fixed by drift, but any changes to a flexible linker that alters the activity of the gene product to produce an advantageous variant would be fixed by natural selection. Thus, regardless of the mechanism of most indel mutations, there are clear reasons to examine the impact of specific indels upon the function of the inhibin α-subunit.

Taken as a whole, our phylogenomic analyses emphasize that the inhibin α-subunit lineage arose by an ancient β-subunit duplication, probably early in deuterostome evolution. Our analyses also indicate that there have been multiple duplications of genes encoding the β-subunit and few (if any) duplications of α-subunit encoding genes. Some of the most striking changes during α-subunit evolution have been large insertions, making it reasonable to postulate that the differences in biological activity between the α-subunit and the activin β-subunits and other TGFβ ligands reflect these insertions.

### Forward-Engineered β_A_-Subunits with α-Subunit Indels Determine Homodimerization Potential

**Figure 3:**
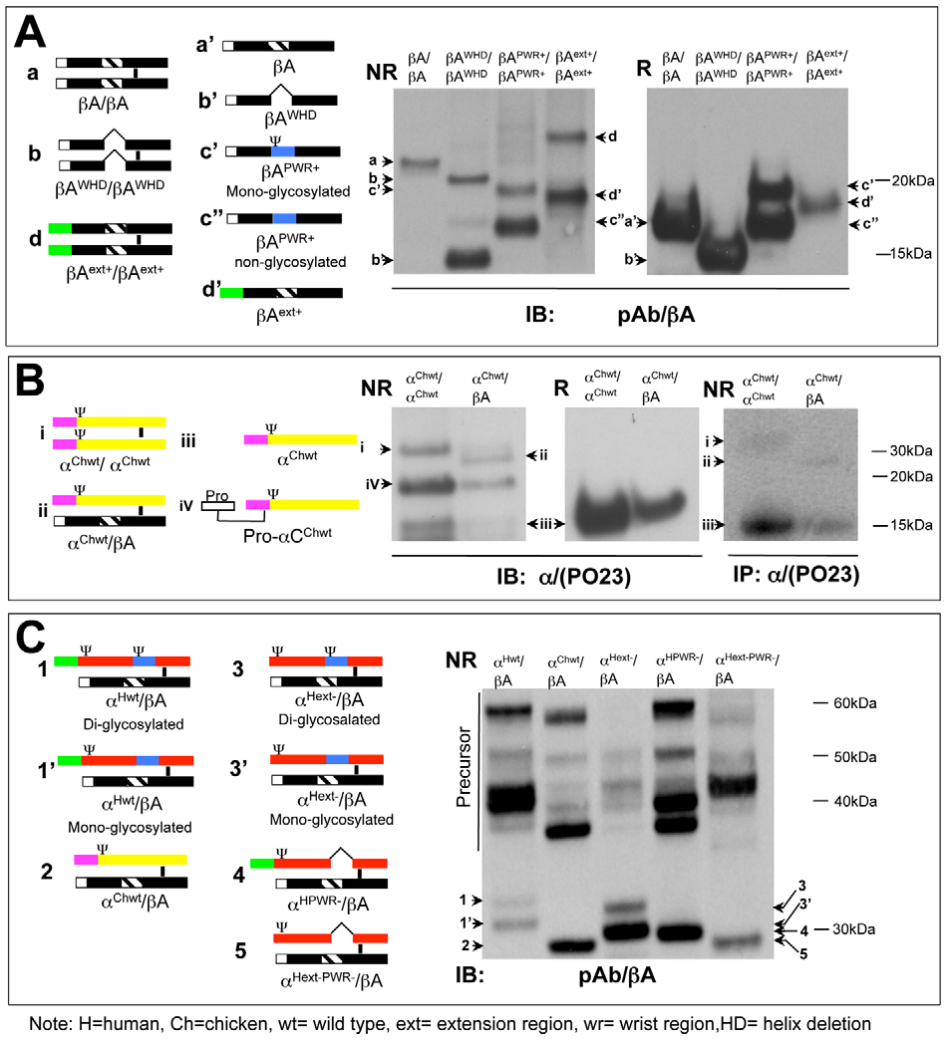
Cloning and expression of engineered inhibin and activin mutants and their wild type molecules. Schematic representations of wild type and mutant inhibin/activin molecules along with immunoblots demonstrating their presence in conditioned media from stably transfected cells. The construct transfected is marked above each lane and the product bands are indicated on the side with lower case letters or numbers or Greece letters that correspond to the schematic representation on the left. N-linked glycosylation site is denoted by the ψ symbol. Details on the design and nomenclature of all the mutants described herein are provided in **Fig. S4** and **Table S3**. **A**) Left, schematic representations of dimeric and monomeric forms of wild type activin (βA/βA), activin chimera mutants (βA^ext+^/βA^ext+^ or βA^PWR+^/βA^PWR+^) and activin deletion mutant (βA^WHD^/βA^WHD^). Right, immunoblot of wild type activin and its chimera and deletion mutants using anti-βA-subunit polyclonal antibody under non-reducing (NR) and reducing (R) conditions. **B**) Left, schematic representations of wild type chicken inhibin (α^Chwt^/βA) as well as dimeric and monomeric forms of chicken free α-subunit (α^Chwt^/α^Chwt^, α^Chwt^ and Pro-α^Chwt^). Middle, immunoblot of wild type chicken free α-subunit and chicken inhibin using anti-human-α-subunit PO23 monoclonal antibody under non-reducing (NR) or reducing (R) conditions. Far Right, non-reducing SDS-PAGE autoradiograph showing the immunoprecipitation (using anti-α-subunit monoclonal antibody PO23) of [^35^S]-cysteine-labeled chicken free α-subunit and chicken inhibin A. Controls for the immunoprecipitation experiment are shown in **Fig. S5A**. **C**) Left, schematic representations of human wild type inhibin A (α^Hwt^/βA), chicken wild type inhibin (α^Chwt^/βA), and three deletion mutants of the human α-subunit (α^Hext−^/βA, α^HPRW−^/βA and α^Hext-PRW−^/βA). Right, immunoblot of wild type human and chicken inhibin A and the human α-subunit deletion mutants using anti-α-subunit PO23 monoclonal antibody under non-reducing (NR) condition. Subsequent immunoblots detected with either anti-α-subunit monoclonal antibody PO23 or anti-β_A_-subunit polyclonal antibody under reducing conditions are shown in **Fig. S6**.

To test the hypothesis suggested by our phylogenomic analyses—that insertions played a major role in the origin of α-subunit function after the α-subunit arose by duplication of another TGFβ superfamily member (β-subunit gene)—we constructed chimeric inhibin/activin genes and examined their biological activity. We modeled the iterative evolution of the inhibin α-subunit using the human β_A_-subunit as a template. Human β_A_-subunits were used because they exhibit more than 90% sequence identity to other vertebrates in the mature region [21], [54], are well-characterized functionally [55], [56], [57] and appear to have undergone limited change since the common ancestor of the inhibin/activin family (Fig. 2C). Forward-engineered β_A_-subunit mutants were constructed to change the β_A_-subunit into an “α-like” subunit. Specifically, we swapped the human α-subunit N-terminal extension (ext) to the β_A_-subunit (βA^ext+^/βA^ext+^ mutant), deleted the β_A_-subunit wrist helix (WH) region (βA^WHD^/βA^WHD^ mutant) and swapped the α-subunit proline-rich wrist region (PWR) into the wrist helix region of the β_A_-subunit (βA^PWR+^/βA^PWR+^ mutant) (Figs. 3A and S4, Table S3). The β_A_-subunit wrist helix region deletion mutant activin (βA^WHD^/βA^WHD^ mutant) retained its ability to homodimerize but some monomer βA^WHD^ product was present (Fig. 3A). The addition of the α-subunit N-terminal extension region to the β_A_-subunit also partially blocked homodimer assembly; however, the addition of the mammalian α-subunit proline-rich wrist region into the β_A_ helix region completely blocked dimer assembly (Fig. 3A). These data are consistent with a model in which early vertebrate inhibin α-like subunits, which have the wrist helix region deletion but lack the proline-rich wrist region insertion, gained some potential for heterodimerization. Addition of the full N-terminal extension then further reduced homodimerization, while facilitating inhibin α-subunit heterodimerization with the β-subunit. With the proline-rich wrist region insertion in the mammalian α-subunit, the α-subunit was then only able to form heterodimers.

### Does Avian Inhibin α-Subunit Retain the Ability to Homodimerize?

Since homodimerization was strongly suppressed when the α-subunit proline-rich wrist region was swapped into the human β_A_-subunit, we asked whether wild-type avian α-subunit (which lacks 4 amino acids in the N-terminal insertion and has no proline-rich wrist region insertion) was able to homodimerize (Fig. S2D). We cloned the avian (chicken α^Chwt^) and mammalian (human α^Hwt^) inhibin α-subunits and expressed each species alone or in a bicistronic vector containing the conserved human β_A_-subunit (α^Chwt^/βA or α^Hwt^/βA) (Figs. 3B and 3C). As expected, α-α homodimers were not detected in a cell line expressing only the mammalian α-subunit [^35^S] labeling of cellular proteins (Fig. S5A). Moreover, the heterodimer was the dominant ligand expressed by the isogenic cell line expressing the avian α-subunit and β_A_-subunit cassette (Figs. 3B and 3C). If the avian (chicken) α-subunit alone was expressed, low, but detectable, α^Chwt^/α^Chwt^ mature homodimers were detected both by immunoblot and autoradiography following [^35^S] labeling of cellular proteins (Fig. 3B and S5A), and were confirmed by MS-MS (Figs. S5B and S5C). These data are consistent with the notion that the ancestral form of the inhibin α-subunit retained some ability to homodimerize, although the dominant secreted form was a heterodimer [58]. Both the N-terminal insertion and the proline-rich wrist region insertion evolved iteratively (shorter insertions are present outside of the eutherians; Figs. 2A and 2C) and the homodimerization potential of the α-subunit appears to have been progressively reduced as the insertions lengthened.

To determine whether the N-terminal extension or proline-rich wrist region insertions present in the human inhibin α-subunit affect α-β heterodimer formation, the relevant mutants were cloned into a bicistronic vector containing the β_A_-subunit. The mutants tested were those relevant to our evolutionary model, including a deletion of N-terminal insertion (α^Hext−^/βA mutant, where amino acids 6–27 have been deleted), a deletion of the proline-rich wrist region insertion (α^HPWR−^/βA mutant, where amino acids 68–85 have been deleted) or in tandem deletion (α^Hext-PWR−^/βA mutant) (Fig. S4, Table S3). There were no significant differences between the inhibin α-subunit deletion mutants and wild-type human inhibin in terms of heterodimer secretion (Figs. 3C and S6).

### Forward-Engineered β_A_-Subunit Indels Regulate Agonist Function

**Figure 4:**
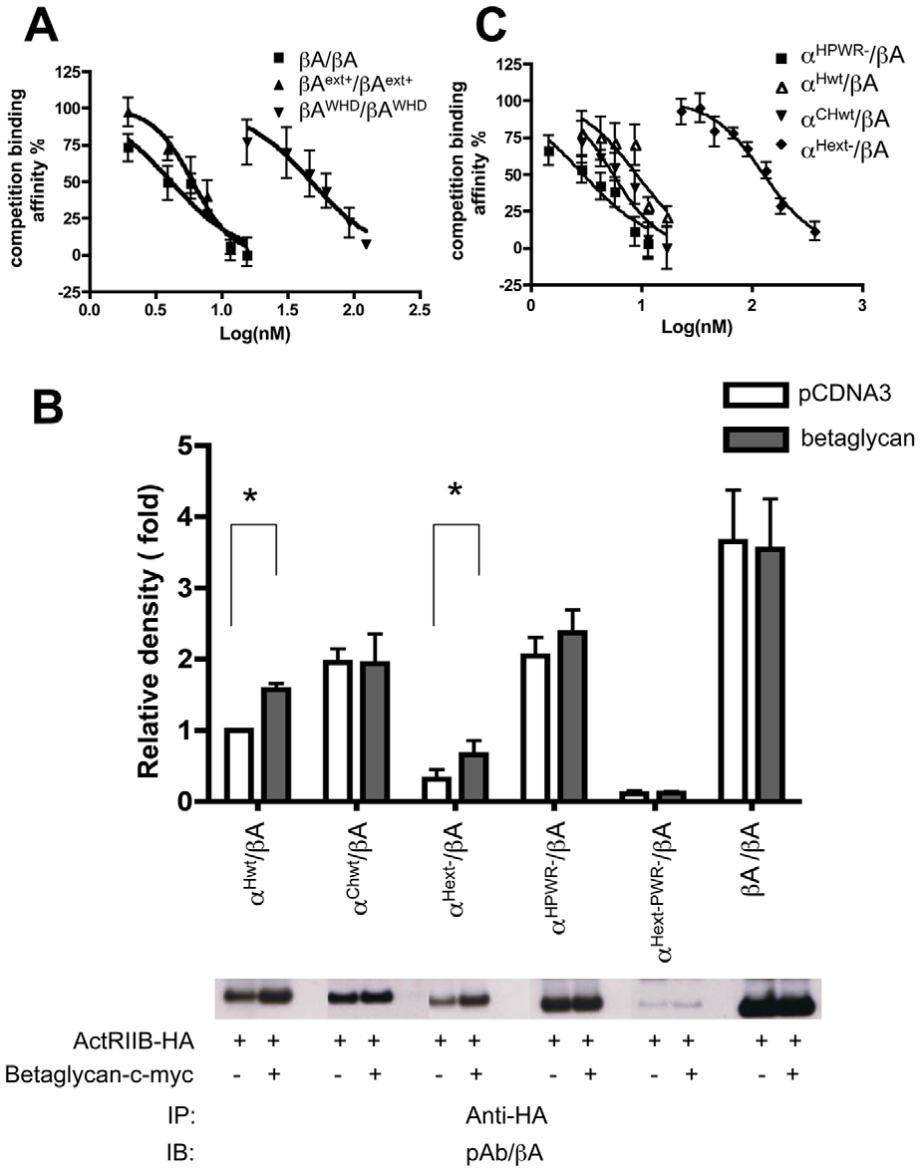
Equilibrium binding of activin and inhibin mutants to ActRIIB using competitive ELISA and immunoprecipitation studies. **A**) Conditioned media from βA/βA, βA^ext+^/βA^ext+^, and βA^WHD^/βA^WHD^ expressing cells were quantified and used to compete with biotinylated activin A for binding to ActRIIB. The IC_50_ for the βA/βA, βA^ext+^/βA^ext+^ and βA^WHD^/βA^WHD^ competition binding curves were 4.14±31.61 nM, 5.99±0.98 nM, 47.6±14.3 nM, respectively, representing a significant difference between all groups as determined by sigmoidal dose-response (variable slope) curve, followed by an F-test (*p*<0.05). The standard curve for biotinylated activin A binding to the ActRIIB (EC_50_ = 1.1 nM) is shown in **Fig. S7A**. **B**) Top, densitometric analysis of immunoblots (bottom) from three independent experiments, Density was normalized to COS7 cells transfected with empty vector (pcDNA3) and treated with human wild type inhibin A. Asterisks represent statistically significant differences using unpaired t-test (*p*<0.05). Bottom, the immunoblots of the immunoprecipitation that use monoclonal anti-HA pulldown for lysates from COS-7 cells transfected with ActRIIB-HA and either full-length betaglycan or empty vector (pcDNA3). Prior to cell lysis, the cells were treated with inhibin deletion mutants, wild type human inhibin A, chicken inhibin A or activin A culture media for 2 hours. The western-blots are detected by anti-β_A_-subunit polyclonal antibody. Controls for the immunoprecipitation studies are shown in **Fig. S8**. **C**) Conditioned media from α^Hwt^/βA, α^CHwt^/βA, α^Hext−^/βA and α^Hwr−^/βA expressing cells were quantified and used to compete with biotinylated inhibin A for binding to ActRIIB. The IC_50_ for the α^Hwt^/βA, α^CHwt^/βA, α^Hwr−^/βA and α^Hext−^/βA competition binding curves were 9.01±3.72 nM, 5.55±1.85 nM, 2.96±1.23 nM and 127.9±17.80 nM, respectively, representing a significant difference between all groups as determined by sigmoidal dose-response (variable slope) curve, followed by F-test (*P*<0.05). The standard curve for biotinylated inhibin A binding to the ActRIIB (EC_50_ = 2.73 nM) is shown in **Fig. S7B**. The graphs shown in (**A**) and (**C**) represent the results of more than three independent experiments.

We next asked whether the forward-engineered activin molecules (dimers of β_A_-subunits containing α-subunit segments) acted as agonists or antagonists. We tested the same three mutant classes created by altering the β_A_-subunit in a manner consistent with the major evolutionary changes identified in the phylogenomic analysis. Again, these changes were an addition of the N-terminal insertion (βA^ext+^/βA^ext+^ mutant), deletion of the wrist helix region (βA^WHD^/βA^WHD^ mutant) and addition of the proline-rich α-subunit wrist region insertion (βA^PWR+^/βA^PWR+^ mutant) (Fig. S4, Table S3). Each mutant was tested in a functional assay that measured FSHβ-luciferase activity in a stably transfected pituitary gonadotrope cell line (−338 FSHβ-Luc) [59]. Addition of the N-terminal insertion reduced the bioactivity of the mutant activin compared to wild type, but still acted as an agonist (mutant EC_50_ = 3.82 nM; wild type EC_50_ = 1.21 nM) (Table 1). Activity of the mutant βA^ext+^/βA^ext+^ dimer was not altered with the addition of the co-receptor betaglycan (EC_50_ = 5.13 nM) (Table 1). In sharp contrast, elimination of the wrist helix region of the activin β-subunits (βA^WHD^/βA^WHD^ mutant) also eliminated activin agonist function (Table 2) [23], [46]; indeed, this ligand showed weak antagonist properties (IC_50_ = 56.7 vs. wild type inhibin A IC_50_ = 1.01 nM) (Table 1). We measured the affinities of wild type activin A, chimera mutant βA^ext+^/βA^ext+^ and deletion mutant βA^WHD^/βA^WHD^ for ActRIIB directly using a competition binding analysis, which revealed a weaker binding constant for the N-terminal insertion mutant and wrist helix deletion mutant (βA^ext+^/βA^ext+^ IC_50_ = 5.99 nM; βA^WHD^/βA^WHD^ IC_50_ = 47.6 nM; human activin A IC_50_ = 4.14 nM) (Figs. 4A and S7A). Thus, the critical switch of the α-subunit from an agonist to an antagonist appears to have coincided with the deletion of the wrist helix region; when examined in isolation, the N-terminal insertion only changed the relative strength of β_A_-subunit agonist activity.

**Table 1:** Functional assay of wild type and mutant inhibin and activin constructs as measured by FSHβ promoter activity in stably transfected LβT2 cells.

Inhibin A/Activin A wild type and mutants	n	IC_50_ (nM)		EC_50_ (nM)		F-test *p*
		−BG	+BG	−BG	+BG	
α^Hwt^/βA antagonist	12	1.01±0.27^a^	0.24±0.03	-	-	<0.05
α^Chwt^/βA strong antagonist	12	0.57±0.25^ab^	0.36±0.33	-	-	>0.05
α^Hext−^/βA weak antagonist	12	4.46±2.26^c^	0.63±0.23	-	-	<0.05
α^HPWR−^/βA strong antagonist	12	0.31±0.16^b^	0.27±0.14	-	-	>0.05
α^Hext-PWR−^/βA not an antagonist	12	-	-	-	-	
βA/βA agonist	9	-	-	1.21±0.16^A^		
βA^WHD^/βA^WHD^ weak antagonist	9	56.7±17.9^d^	-	-	-	
βA^ext+^/βA^ext+^ weak agonist	9	-	-	3.82±0.33^B^	5.13±2.23	>0.05
βA^PWR+^/βA^PWR+^ poor dimerization	-	-	-	-	-	

IC_50_: concentration of ligand causing 50% of the maximum inhibition.

EC_50_: concentration of an agonist producing 50% of the maximum possible response.

a,b,c,d represents differences between the groups, *p*<0.05 by F-test.

A,B represents differences between the groups, *p*<0.05 by F-test.

BG  =  betaglycan, H  =  human, Ch  =  chicken, wt  =  wild type.

ext  =  N-terminal extension, PWR  =  Proline-rich wrist region, WHD  =  wrist helix deletion.

**Table 2:** Summary of wild type and engineered inhibin/activin indel mutant.

	Mature dimer processing	Activin-like function	Inhibin-like function	Affected by betaglycan
α^Hwt^/βA	Yes	No	Yes	Yes
α^Chwt^/βA	Yes	No	Yes, but stronger than human wild type inhibinA	No
α^Hext−^/βA	Yes	No	Yes, but weaker than human wild type inhibinA	Yes
α^HPWR−^/βA	Yes	No	Yes, but stronger than human wild type inhibinA	No
α^Hext-PWR−^/βA	Yes	No	No	No
βA/βA	Yes	Yes	No	No
βA^WHD^/βA^WHD^	Yes	No	Yes, but weaker than human wild type inhibinA	No
βA^ext+^/βA^ext+^	Yes	Yes, but less than wild type activinA	No	No
βA^PWR+^/βA^PWR+^	Very little	No	ND	ND

ND  =  not determined.

As expected based upon our model of inhibin α-subunit evolution, placement of the proline-rich wrist region of eutherian mammals into the βA-helix region (βA^PWR+^/βA^PWR+^ mutant) does not restore the agonist function of the βA^WHD^/βA^WHD^ mutant (Table 2). However, βA^PWR+^/βA^PWR+^ mutants also did not form productive homodimers (Fig. 3A). Taken as a whole, these data indicate that deletion of the wrist helix region was a critical step for functionally switching the β-subunit from an agonist to antagonist. Based upon our phylogenomic analyses of the inhibin/activin gene family, we postulated that the α-subunit arose by neo-functionalization after duplication a β-subunit gene; these analyses indicate the mutation most likely to be responsible for this change is the deletion of the wrist helix region.

### Reverse-Engineered α-Subunit Indels Identify Functional Changes

Because non-mammalian inhibin α-subunits (including those from fish, amphibians, reptiles and birds) contain the wrist helix deletion typical of all non-mammalian inhibin α-subunits but lack the proline-rich wrist region insertion found in mammals (Figs. 2A and 2C), we wanted to examine the activity of a non-mammalian α-subunit. The chicken inhibin (α^Chwt^/βA) was chosen as a representative non-mammalian α-subunit to test in the FSHβ functional assay (since the activin type II receptor and betaglycan ZP domain are conserved between the avian and mammal species) for comparison to human inhibin A (α^Hwt^/βA). We observed two notable differences between the functional capacity of human inhibin and chicken inhibin. First, chicken inhibin was a slightly more potent antagonist than human inhibin (chicken inhibin A IC_50_ = 0.57 nM; human inhibin A IC_50_ = 1.01 nM) (Table 1). Second, while the co-receptor betaglycan enhanced human inhibin activity (IC_50_ = 0.24 nM) as expected [11], [60], addition of betaglycan did not further affect the already high chicken inhibin antagonist function (IC_50_ = 0.36 nM) (Table 1).

Interaction of human and chicken inhibins with the ActRIIB activin receptor was then examined by co-immunoprecipitation. Whereas chicken inhibin interacted with ActRIIB similarly in the presence and absence of betaglycan, human inhibin binding to ActRIIB was enhanced by betaglycan (Figs. 4B and S8). We measured the affinities of chicken and human inhibin for ActRIIB directly using a competition binding analysis, which revealed a stronger binding constant for chicken inhibin (IC_50_ = 5.55 nM vs. human inhibin A IC_50_ = 9.01 nM) (Figs. 4C and S7B). Thus, the ancestral α-subunit could have been a slightly stronger antagonist with a higher affinity to the ActRIIB that was able to act in a betaglycan-independent manner to block activin action.

Because none of the deletion mutants exhibited problems with heterodimer formation, we had the opportunity to further test the impact of specific mutations on inhibin function. We first tested deletion of the proline-rich wrist region insertion in the human α-subunit (α^HPWR−^/βA mutant), which revealed that this mutation creates a remarkably potent antagonist that no longer requires betaglycan (IC_50_ = 0.27 nM with betaglycan vs. IC_50_ = 0.31 nM without betaglycan) (Table 1). Moreover, this mutant behaved similar to chicken inhibin in co-immunoprecipitation assays with ActRIIB and betaglycan (i.e., there was little difference in α^HPWR−^/βA mutant inhibin binding to ActRIIB with or without betaglycan; Figs. 4B and S8). ActRIIB receptor interaction was at a very high affinity (α^HPWR−^/βA IC_50_ = 2.96 nM vs. wild type human inhibin A IC_50_ = 9.01 nM) (Figs. 4C and S7B), consistent with strong antagonistic function in the FSH bioassay.

Deletion of the N-terminal insertion (α^Hext−^/βA mutant) resulted in an inhibin that was less bioactive than wild type inhibin (IC_50_ = 4.46 nM) (Table 1). This result was consistent with previous findings that an antibody to the N-terminal extension region bioneutralized inhibin function [24], [25]. The N-terminal deletion mutant showed increased antagonist function with the addition of betaglycan, similar to wild type human inhibin (IC_50_ = 0.63 nM with betaglycan) (Table 1). The N-terminal deletion mutant bound to ActRIIB in the co-immunoprecipitation assay, which increased with the addition of betaglycan (Figs. 4B and S8). The competition binding affinity of the N-terminal deletion mutant to ActRIIB was weaker than that observed for human wild type inhibin A (IC_50_ = 127.9 nM) (Figs. 4C and S7B). Elimination of the both the N-terminal insertion and the proline-rich wrist region insertion (α^Hext-PWR−^/βA mutant) resulted in the almost complete abolition of inhibin bioactivity, and no activin A-like agonist function. Betaglycan did not rescue the antagonist function of this double mutant (Table 2). The double deletion mutant inhibin A exhibited no binding to ActRIIB, with or without betaglycan, similar to the empty vector (Figs. 4B and S8). This mutant did not show any competition binding affinity to ActRIIB with biotinylated human wild type inhibin A (data not shown).

Taken as a whole, these experiments emphasize that the proline-rich insertions found in the inhibin α-subunit of eutherian mammals resulted in profound changes to the biological activity of that gene. Specifically, loss of the wrist region helix from the β-subunit early in the vertebrate evolution, probably prior to the vertebrate WGD but after the divergence of vertebrates and cephalochordates, resulted in a shift from an agonist to an antagonist. The insertion of a long proline-rich sequence into the area, where the wrist region helix had been located, abolished any remaining homodimerization potential and allowed interaction with the betaglycan co-receptor. The insertion in the N-terminal region also had an impact on homodimerization capacity and the activity of the dimer, but functional changes related to insertions in this region appear to have been more limited in the vertebrate inhibin α-subunit. Nonetheless, these experiments strongly confirm the predictions of the phylogenomic study that indels would prove to be central to functional changes among members of the inhibin/activin family.

## Discussion

### Inhibin and the Progressive Evolution of Vertebrate Reproductive Strategies

Inhibin is the only known endocrine hormone in the TGFβ superfamily, and based on the present phylogenomic analysis, likely arose early in vertebrate evolution, before the WGD that characterizes the vertebrate genome. Inhibin plays an essential role in the negative feedback between the gonads and the pituitary gland that are necessary for the regulation of reproductive function [17], [61]. FSH in the pituitary is stimulated by locally produced activin, and ovarian-derived inhibin antagonizes activin in the pituitary [14], [15], [17]. Interestingly, the inhibin α-subunit and the β-subunit of FSH might have arisen at similar times; both molecules arose by gene duplications early in vertebrate evolution [62], and are necessary for the integration of information from distal tissues to control reproduction.

**Figure 5:**
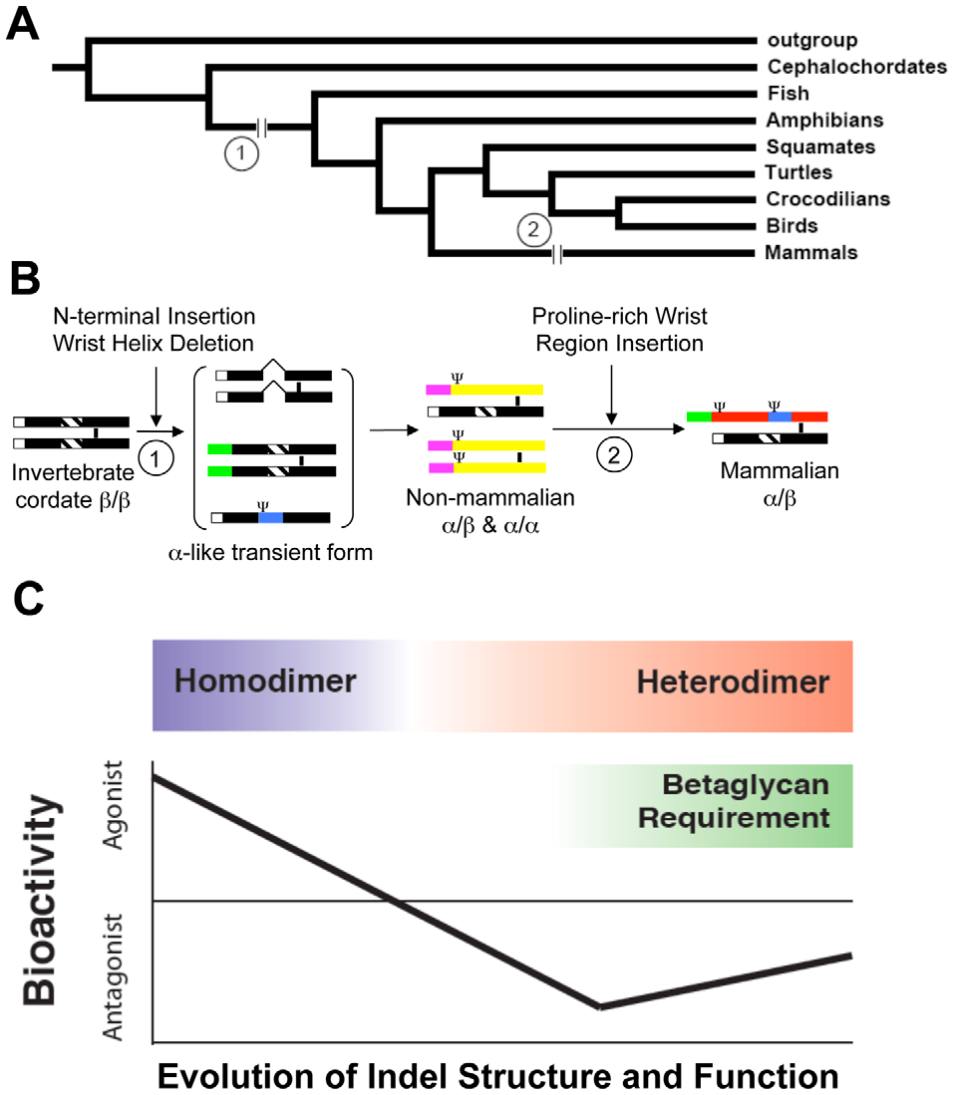
Schematic diagrams illustrating the proposed model for the evolution of the inhibin α-subunit. **A**) Indels that have had a major impact upon α-subunit function are indicated on the deuterostome tree. Two major indel changes (the N-terminal insertion and wrist helix region deletion [1] and the proline-rich wrist region insertion [2]) occurred at distinct times in evolution. **B**) Schematic representation of the evolution of inhibin α-subunit. The graph includes the forward engineered mutants used in our experiment that are representative of α-like transient forms. Non-mammalian α-subunit structure is represented by the chicken α orthologue, and mammalian α-subunit structure is diagramed by the human α orthologue. **C**) Evolution from inhibin β-subunit to inhibin α-subunit involves the loss of the ability to homodimerize. Concurrent with this evolution was a change in the bioactivity from agonist to strong antagonist that is betaglycan independent, and later to weak antagonist that requires betaglycan for maximal antagonistic function. The indel in the evolution of inhibin α-subunit strongly affects inhibin function.

To learn more about inhibin and provide clues about its origins and iterative changes that resulted in the creation of a powerful endocrine antagonist within the TGFβ family and the reproductive axis, we used phylogenomics in combination with rationally designed mutants that were tested in well-characterized functional and binding assays (Figs. 5A and 5B). We learned two important pieces of information from this work. First, the β-subunit-like ancestor of the inhibin α-subunit underwent at least two key changes that resulted in the acquisition of novel functions (neo-functionalization) and led to the emergence of the inhibin α-subunit (Fig. 5A). Second, a hierarchy of functions can be assigned to specific molecular signatures (the indels) that allow us to understand the changes that occurred during the evolution of this molecule (Fig. 5C).

Our data are consistent with a model in which the invertebrate β-subunit underwent a series of changes to become a functional α-subunit. The lancelet α-subunit-like sequence, united with vertebrate α-subunit and, based upon the results of phylogenomic analyses (Figs. 2C and S3), has a partial deletion of the helix and a distinct N-terminal extension, suggesting it may be a “missing link” between the α- and β-subunits. Loss of the helix region present in the β-subunits, which is responsible for receptor binding, particularly to the activin type I receptor (ALK4) [10], [45], [46], switched the function of the molecule from an agonist to an antagonist and facilitated heterodimer formation. Loss of the helix region alone, however, was not sufficient to achieve full α-subunit functionality; the N-terminal extension developed in parallel to strengthen the antagonistic properties of the α-subunit and further favor heterodimer formation (Fig. 5).

The inhibin α-subunit in non-mammalian vertebrates has a deletion of the wrist region (e.g., the chicken α-subunit) and inhibin dimers containing these α-subunits do not require the accessory protein betaglycan. As the wrist region became populated by a series of proline amino acids, likely to have first appeared as short insertions in the common ancestor of monotreme-therians and later expanded to form larger insertions in metatherian and eutherian mammals, the inhibin molecule was able to better coordinate betaglycan receptor binding (Fig. 5C). We predict that most non-mammalian vertebrates exhibit a more powerful betaglycan-independent antagonism similar to that exhibited by avian inhibin. By contrast, mammalian inhibin is a relatively weaker antagonist of activin signaling that is dependent upon betaglycan as a co-receptor (Fig. 5C). Inhibin has a role limited to the time of sexual maturity in birds and progesterone regulates pituitary function after that time. Indeed, immunoneutralization of chicken inhibin brings the animals into egg-laying earlier [63] and the birds continue to cycle after entering sexual maturity. Thus, a strong endocrine antagonist is necessary for the onset of normal sexual maturity in the chicken, but is not necessary in the normal adult cycle [64], [65], [66]. Conversely, immunoneutralization of mammalian inhibin causes a prompt rise in FSH and the end of adult cyclicity [25], [67]. Thus, a weaker antagonist may be necessary to ensure an on-and-off rate necessary for a repetitive cycle that must be reset rapidly (up to every four days in some rodents) [68].

A fundamental question in evolutionary biology is whether innovations (or novelties) can be accommodated within the neo-Darwinian framework [69], [70]. There has been vigorous debate regarding the roles of drift and selection in the evolution of genes, genomes and organisms [71], [72], but the mechanisms by which genomic changes result in evolutionary novelties remain unclear. The evolution of inhibin, a TGFβ ligand that can act as an endocrine hormone, represents a potentially important molecular innovation. The molecular basis of this innovation appears to be gene duplication followed by a sequence change that inhibits homodimerization (Fig. 5C), a process that conforms to one of the well-established “principles governing molecular evolution” [73]. The origin of novel functions after gene duplication is easily accommodated within modern evolutionary theory [74], [75], [76]. This is true even for some of the more recently suggested pathways for the origin and preservation of duplicate genes [74], [75], [77], [78]. Ultimately, changes to a duplicate gene that appear to conform to expected types of change [73] led to the advent of the inhibin α-subunit which resulted in an innovation that allowed communication between gonads and pituitary, itself a novelty at the level of the whole organism. This communication between the gonads and pituitary represents the evolution of an important endocrine feedback mechanism, perhaps the first negative feedback loop to emerge in vertebrate animals.

Here we have presented evidence regarding the nature of specific evolutionary changes that led to the origin of the inhibin α-subunit in the vertebrate lineage. These molecular changes appear to result in innovations, both in molecular function and in the specialization of the reproductive axis, specifically the ability to act as an endocrine antagonist. The subsequent molecular changes leading to a “mammalian” mode of inhibin signaling also emphasized the role of co-option or recruitment in genetic innovation. Specifically, the wrist region insertion resulted in the recruitment of betaglycan, which had an established role in TGFβ signaling [60], [79], [80], to enhance the inhibin signaling system. Our experiments indicate that well-established processes such as gene duplication followed by sequence changes can explain these evolutionary novelties. However, a striking difference between the most prominent sequence changes noted in this study of inhibin evolution and those noted in many other studies is the importance of indels rather than amino acid substitutions [10]. In contrast to the excellent methods available to examine positive selection for amino acid sequences [81], [82] that have been successfully applied at a genomic scale [82], [83] tools that can be used to examine the potential functional impact of indels remain poorly developed. Despite the relative paucity of analytical tools, it is clear that indels make an important contribution to genomic divergence [84] and to changes in protein function [83], [85], [86]. Our results are striking in that they directly implicate a relatively poorly characterized type of sequence change in the origin of inhibin function. Nonetheless, the fact that the impact of indels on protein function remains poorly characterized relative to the impact of amino acid substitutions does not a change the fact that indels can be accommodated within the neo-Darwinian paradigm.

Development of the inhibin α-subunit resulted in the first negative feedback peptide-hormone based endocrine system in animals by providing a circulating, gonadally-derived antagonist to the pituitary-made activin. Neo-functionalization of the inhibin α-subunit expanded the possibilities for animal reproductive strategies. The early α-subunits, characterized by N-terminal insertion and the wrist region deletion were able to block activin receptors without an accessory protein, although they retained some ability to homodimerize. The wrist region underwent additional insertions in the mammals that led to further functional modification, including the inability to homodimerize and a requirement for the betaglycan co-receptor, further attesting to the importance of this feedback loop for animal reproductive strategies. Combining phylogenomic analysis together with functional assays of a rationally designed set of mutant proteins provided a remarkable opportunity to understand the origins of the evolutionary complexity of the inhibin system and provide information about the reproductive evolutionary changes needed to create this agonist-antagonist pair.

## Materials and Methods

### Gene Sequencing and Phylogenomic Analysis

A diverse set of TGFβ homologues were retrieved from complete genome sequences (downloaded from NCBI [http://www.ncbi.nlm.nih.gov] and the DOE Joint Genome Institute [http://www.jgi.doe.gov]) using BLASTP searches [87]. Initial sequence alignments were obtained using MAFFT [88], [89] and optimized by eyes using the conserved cysteine residues, the conserved W-X-X-W motif and conversed RXXR proconvertase enzyme cleavage site. To examine the phylogenomic distribution of specific α-subunit indels, additional sequences of the mature inhibin α-subunit were determined by RT-PCR or PCR of genomic DNA. It was reasonable to use genomic DNA as a template because the α-subunit comprises two exons in all species studied to date and the splice sites are found upstream of the region encoding the N-terminus of the mature region. The ML estimate of phylogeny was obtained using PhyML 3.0 [89] using the JTT model [90] of amino acid evolution with the rates at different sites drawn from a Γ-distribution, which was judged to be the best-fitting model using standard criteria [41]. Support for specific groups was assessed using 100 bootstrap replicates and indels were mapped onto the phylogeny using the maximum parsimony criterion [91], [92].

### Mutagenesis and Protein Production

Activin β_A_-subunit chimeras containing regions of the inhibin α-subunit were produced by amplifying appropriate regions of the inhibin α-subunit (the N-terminal insertion and the proline-rich wrist region insertion) using primers containing partial activin β_A_-subunit and partial inhibin α-subunit sequences. The QuikChange procedure (Stratagene, La Jolla, California, USA) was then followed using the amplicons as the primers in a PCR reaction in which a pcDNA3 plasmid containing the βA gene was the template. Inhibin deletion mutants were also made with the QuikChange method using primers spanning, but not including, the region of the α-subunit that was removed (Table S3). The template for the reaction was a pcDNA5/FRT bicistronic plasmid containing the human α- and β_A_-subunits separated by an internal ribosomal entry site [37]. The full-length chicken α-subunit cDNA was kindly provided by Dr. Patricia A. Johnson (Cornell University, Ithaca, New York, USA) and subcloned into the first multiple cloning site of the inhibin A bicistronic plasmid replacing the human α-subunit cDNA. Either full-length human or chicken α-subunit was subcloned into pcDNA5/FRT. Mutations were verified by sequencing in an ABI3100 Capillary DNA Sequencer. CHO cells were transfected with the activin chimera mutants using Lipofectamine 2000 (Invitrogen, Carlsbad, California, USA). Transfected CHO cells were selected using G418 antibiotic (500 µg/ml) and carried in DMEM-F12 media supplemented with 5% fetal bovine serum, 1% penicillin/streptomycin. CHO Flp cells were transfected with inhibin deletion mutant plasmids or inhibin free α-subunit plasmids with pOG44 plasmid and were selected using hygromycin B antibiotic (500 µg/ml) and carried in F12 media supplemented with 10% FBS and 1% penicillin/streptomycin. Three isogenic cell lines were generated for human inhibin A, chicken inhibin A, human inhibin free α-subunit, chicken inhibin free α-subunit and each inhibin deletion mutant [37]. At confluence, media was exchanged for DMEM-F12 or F-12 serum-free media and grown for 3–4 days before collection. Following collection, serum-free media was dialyzed into 50 mM Tris, 150 mM NaCl, and the protein solution was concentrated by Amicon Ultra Centrifugal Filter Units (Millipore, Billerica, MA, USA) [10]. The concentrated media was directly used in the bioassay and binding assay.

### Immunoblot Analysis of Ligands and Protein Concentration Determination

Wild type, chimera mutant, and human α-subunit deletion mutant ligands were electrophoresed on 4–12% SDS-PAGE gels under non-reducing conditions and transferred to nitrocellulose for immunoblot analysis. The ligands were detected using a polyclonal antibody to the inhibin β_A_-subunit antibody (kindly provided by Dr. W. Vale, The Salk Institute, La Jolla, California, USA) followed by horseradish peroxidase-conjugated anti-rabbit secondary antibody (Zymed, San Francisco, California, USA). The secondary antibody was detected using ECL (Pierce Chemical Co., Rockford, Illinois, USA) and exposed at varying time points to X-ray film (Kodak, Rochester, New York, USA). Densitometric analysis of signals was quantified using the KODAK 4000 MM Digital Imaging System and analyzed using Kodak Imaging Software (version 4.0.1 Kodak, Rochester, NY) [37]. Purified human wild type activin A and inhibin A of known concentration (from our laboratory) was used as a standard to quantify the amount of ligand from wild type activin A or inhibin A and mutants culture media concentrated and dialyzed under non-reducing conditions. The inhibin A deletion mutant and activin chimera mutants were detected using one of three antibodies to the mature α-subunit: anti-α-subunit R1 monoclonal antibody (from Serotec, Raleigh, North Carolina, USA) directed toward the extended region, anti-α-subunit PO14 monoclonal antibody (provided by Dr. David Phillips, Monash Institute, Australia) directed toward the wrist region, and anti-α-subunit PO23 monoclonal antibody (provided by Dr. David Phillips, Monash Institute, Australia) directed toward the C-terminal region (Fig. S1).

### Metabolic In Vivo Labeling and Subunit Immunoprecipitation

Isogenic cultures of cells expressing wild type human inhibin A, chicken inhibin A, human inhibin free α-subunit and chicken inhibin free α-subunit in 12 well plates were continuously cultured in the presence of [^35^S]-cysteine. After 48 hours, media was collected and immunoprecipitated with anti-α-subunit monoclonal antibody PO23 or normal mouse IgG, and then carried with the Protein G Sepharose 4 Fast Flow (GE Healthcare, Sweden). Beads were then boiled for 5 min, centrifuged and the supernatants were analyzed on 4–12% SDS-PAGE gel under non-reducing conditions. Autoradiography using X-ray film (Kodak, Rochester, New York, USA) was conducted for about 7 days.

### Tandem MS-MS Fragment Mass Mapping

Culture media from cells stably expressing the chicken free α-subunit was collected and immunoprecipitated with an anti-α-subunit monoclonal antibody PO23 and subjected to SDS-PAGE under non-reducing conditions. After the gel was stained by Coomassie Blue (Bio-Rad, Hercules, California, USA), the 30 kDa band was excised and sent to the protein and nucleic acid facility at the Stanford University Medical Center (Stanford, California, USA). The band was digested with trypsin and subjected to tandem MS-MS analysis.

### Luciferase Assays of FSHβ Expression

The bioactivity of wild type and chimera proteins was determined using LβT2 gonadotrope cell line stably transfected with the −338 region of the FSHβ promoter conjugated to a luciferase reporter [59]. Cells were cultured as reported previously [59], and treated for 6 hours with various concentrations of wild type activin A or media from cells expressing the activin A chimera mutants, or activin A (0.4 nM) with various concentrations of media from cells expressing wild type human inhibin A, chicken inhibin A, or human inhibin A deletion mutants at 37°C in serum-free DMEM/F12 (1∶1), phenol red-free (Invitrogen, Carlsbad, California, USA), supplemented with 1% penicillin/streptomycin. Following treatment, cells were lysed in GME buffer with 1% Triton-X100 and 1 mM DTT, and then treated with assay buffer (GME buffer, 16.5 mM KPO_4_, pH 7.8 2.2 mM ATP, and 1.1 mM DTT). Luciferase activity was measured for 30 seconds using an AutoLumat luminometer (Berthold Technologies Co., Oak Ridge, Tennessee, USA) [10], [59].

To assess the effect of betaglycan on the bioactivity of each mutant, LβT2 cells were transfected with 0.5 µg of rat betaglycan-c-myc expression plasmid (provided by Dr. Fernando Lopez Casillas, Howard Hughes Medical Institute, Maryland, USA) or 0.5 µg of empty vector pCDNA3.0 using Lipofectamine 2000 (Invitrogen, Carlsbad, California, USA) in 24-well plates. Betaglycan transfection efficiency was tested by immunoblot using monoclonal anti-c-myc antibody. Cells were treated with the appropriate ligand 24 hours after transfection.

### Immunoprecipitation of Receptor Complexes

COS7 cells were utilized for immunoprecipitation experiments. Cells were cultured in DMEM supplemented with 10% fetal bovine serum and 1% penicillin/streptomycin. At confluency, cells were split to 2×10^6^ cells per 60-cm^2^ dish. Twenty-four hours later, cells were transfected with 6 µg of rat ActRIIB-HA expression plasmid (provided by Dr. Kelly Mayo, Northwestern University, Evanston, Illinois, USA) and either 6 µg of empty vector pCDNA3 or mouse betaglycan-c-myc expression plasmid. The next day, normal growth media was added to allow the cells to recover, and after an additional 24 hours, cells were treated with 100 ng/ml of the concentrated media. Immunoprecipitation was carried out as reported previously [10].

### Binding Assay

Purified human inhibin A or activin A was biotinylated using the EZ-link Micro Sulfo-NHS-LC-biotinylation Kit (Pierce, Rockford, Illinois, USA). The binding of biotinylated inhibin A or activin A to activin type IIB receptor (ActRIIB) was measured using an enzyme linked immunosorbent assay (ELISA). 50 ng of ActRIIB in 50 µl of 20 mM HEPES buffer, pH 7.4 was dried overnight in each well of a 96-well microtiter plate (Nunc MaxiSorp™, Fisher Scientific, Pittsburgh, Pennsylvania, USA). Each well was blocked for 30 min with 100 µl of 0.5% BSA in PBS, aspirated and washed with 250 µl of PBS. Each well was then incubated for 1.5 hours with 50 µl of various concentrations of biotinylated inhibin A in TBST (Tris-buffered saline, pH 7.4, containing 0.05% Tween 20) at room temperature with shaking. Wells were aspirated and washed three times with 250 µl of TBS-T buffer. Each well was then incubated for 30 min with 100 µl of a 0.1 µg/ml solution in PBS of streptavidin-coupled horseradish peroxidase (Pierce Chemical Co, Rockford, IL, USA). Wells were aspirated, washed three times and developed for 15 min with 100 µl of 1-step Ultra TMB (3,3′,5,5′-tetramethylbenzidine) obtained from Pierce Chemical Co. (Rockford, IL, USA). Color development was stopped by the addition of 100 µl 2 M sulfuric acid. The plate was read using plate reader (Synergy HT Multi-Mode Microplate Reader, Biotex, Houston, TX, USA) at 450 nm.

### Competition Binding Assay

The competition binding of culture media from cells expressing wild type activin A and activin A/inhibin A chimera mutants to the ActRIIB was carried out using biotinylated activin A (EC_50_ = 1.1 nM based on the biotinylated activin A standard binding curve shown in Fig. S7A). The competition binding of culture media from cells expressing wild type inhibin A, chicken inhibin A, and all of the inhibin α-subunit deletion mutants to the ActRIIB was carried out using biotinylated inhibin A (EC_50_ = 2.73 nM based on the biotinylated inhibin A standard binding curve shown in Fig. S7B). Different concentrations of inhibin A in the media were mixed with biotinylated inhibin A in the binding assay system. The binding assay procedure is the same as described above. The concentration of the mature inhibin A or activin A mutants was calculated based on the immunoblot.

### Statistics

Values are reported as the means ± SD. IC_50_ values were determined using the sigmoidal dose-response (variable slope) curve. Statistical analyses were performed using Prism software (Version 4.0a, GraphPad Software, San Diego, California, USA). The F-test was used for the comparison of the IC_50_ values in each different group. Statistical significance was reported if *p*<0.05. Comparison of the densitometric analysis between different groups was determined using two-tail unpaired t-test. Statistical significance was reported if *p*<0.05.

## Supporting Information

Figure S1 Schematic representation of the human and chicken inhibin α and human βA pre-pro subunits. i, The entire human pro-αN-αC is depicted. The full length unprocessed subunit is 366 amino acids. The mature domain (αC) is contained in the 3′ end of this subunit and is cleaved by the proconvertase enzyme at amino acid 232. The red color shows the human αC domain. The N-terminal extension region of the human α-subunit is shown in green bar, the proline-rich “wrist region” of the human α-subunit is shown in blue bar. ii, The entire chicken pro- αN-αC is depicted. The full length unprocessed subunit is 328 amino acids. The mature domain (αC) is cleaved by the proconvertase enzyme at amino acide 215. The yellow color shows the chicken αC. The N-terminal extension region of the chicken α-subunit is shown in pink bar. iii, The entire human pro-βA is depicted. The full length unprocessed subunit is 426 amino acids. The mature domain (βA) is contained in the 3′ end of this subunit and is cleaved by the proconvertase enzyme at amino acide 310. The black color shows human βA. The white bar represents the N-terminal region of the mature βA-subunit, with the crosshatch pattern bar indicating the wrist α-helical region. The black bars show the antibody binding sites that are indicated for the R1, PO14, and PO23 inhibin α-subunit-specific monoclonal antibodies, as well as for pAb βA. The proconvertase enzymecleavage sites were presented by black arrow. The symbol indicates glycosylation sites.
(0.34 MB PDF)

Figure S2 Large-scale estimate of inhibin/activin phylogeny. A) Maximum likelihood (ML) estimate of phylogeny for genes retrieved from annotated animal genomes by BLAST (Altschul et al., 2009) searches. The alignment included only the TGFβ domain of genes that have human inhibin/activin genes as their top hit (they all satisfied the bidirectional best hit criterion). This analysis was used the WAG (Whelan and Goldman, 2001) model with the a proportion of sites assumed to be invariant and the remaining sites at different rates drawn from a -distribution (with a shape parameter estimated by ML). Support for clades reflects the percentage of 100 bootstrap replicates. B) Alignment used for part A of this figure, with the 3 amino acid insertion that unites the lancelet (Branciostoma) α-subunit-like protein with the vertebrate (in this alignment, human and zebrafish) inhibin α-subunit highlighted using yellow. C) Alignment for the lancelet α-subunit-like protein with the lancelet β protein mature domain sequence. D) Alignment for the mature domain sequences between the human inhibin α-subunit, chicken inhibin α-subunit and human inhibin βA subunit. The red box that used in the C) and D) highlighted the regions that we focused on.
(1.45 MB PDF)

Figure S3 Detailed version of the inhibin/activin phylogeny presented in Fig. 1C. The ML analysis used the JTT+ ζ+invariant sites model, as described in the text. Support for specific groups reflects the percentage of 100 bootstrap replicates (only values ≥50%). The version in the text indicates the occurence of indel mutations, including the indels shown to have a major functional impact.
(0.08 MB PDF)

Figure S4 Detail of mutations within human inhibin αC and βA-subunits. A) The schematic representation of wild-type human αC and human βA-subunits. The letters “A”, “B”. “C”, “D”, “E” and “F” denote the six candidate regions targeted for deletion. B) The residue numbers and sequences for the six regions targeted for deletion and used for generating the inhibin α-subunit deletion mutants and inhibin βA-subunit chimera mutants. C) Design and naming conventions for the α-subunit and β-subunit mutants.
(0.32 MB PDF)

Figure S5 Negative control for the Fig. 3B and Tandem MS-MS fragment mass mapping. A) Left, immunoblot of media from cells expressing wild type human α-subunit alone and human inhibin A under non-reducing or reducing conditions. Middle, immunoblot of media from cells expressing human free α or human inhibin A that were labeled with [35S]-cysteine for 48 hours. Media were collected and immunoprecipitated with an anti-α-subunit monoclonal antibody PO23 and then subjected to SDS-PAGE under non-reducing conditions and autoradiography. Right, negative control immunoblot of mouse IgG-immunoprecipitated [35S]-cysteine labeled media. Labels 1, 2, 3 and 4 indicate media from cells expressing αHwt/αHwt, αHwt/βA, αChwt/αChwt and αChwt, respectively. B) The culture media from the chicken α-subunit alone expression cell was collected and immunoprecipitated with an anti-α-subunit monoclonal antibody PO23 and subjected to SDS-PAGE under non-reducing conditions. The gel was stained with Coomasie. The band marked with “*” is supposed chicken α-α homodimer band The molecular weight is around 30 kDa. C) The band was digested with MS grade trypsin and subjected to tandem MS- MS analysis. Using the SwissProt database, the resulting fragment from the band mapped to the chicken inhibin α-subunit mature domain. The matched fragment is highlighted in red.
(3.97 MB PDF)

Figure S6 Immunoblots of media from cells expressing wild type human and chicken inhibin A and various human α-subunit deletion mutants run under reducing conditions. Labels 1′, 2′, 3′, 4′ and 5′ indicate media from cells expressing αHwt/βA, αChwt/βA, αHext-/βA, αHPWR-/βA and αHext-PWR-/βA, respectively. A) Blot was detected by an anti-α-subunit monoclonal antibody PO23. B) Blot was detected by anti-βA-subunit polyclonal antibody.
(1.58 MB PDF)

Figure S7 Standard curves for biotinylated activin A/inhibin A binding to ActRIIB. A) Standard curve for biotinylated activin A; EC50 = 1.10 nM. Related to Fig. 4A. B) Standard curve for biotinylated inhibin A; EC = 2.73 nM. Related to Fig. 4C.
(0.12 MB PDF)

Figure S8 Negative and positive controls for the co-immunoprecipitation experiments presented in Fig. 4B. COS7 cells were transiently transfected with ActRIIB-HA with either pCDNA3 or betaglycan-c-myc (BG-c-myc). A) Cells were treated with 100 ng/ml human activin A or empty vector culture media (EV media). The cell lysate was immunoprecipitated by monoclonal anti-HA or mouse IgG, followed by immunoblot with anti-βA-subunit polyclonal antibody. B) Cells were treated with 100 ng/ml human activin A (panel 1); empty vector (panel 2), human wild type inhibin A (panel 3); chicken wild type inhibin A (panel 4); or culture media from cells expressing human inhibin A deletion mutants αHext-/βA (panel 5), αHPWR-/βA (panel 6), or αHext-PWR-/βA (panel 7). Proteins were detected in the cell lysates by immunoblotting with either monoclonal anti-c-myc or monoclonal anti-HA antibodies.
(1.56 MB PDF)

Table S1 Species database information for TGFβ family members analyzed in Fig. 1A and Fig. 1B.
(0.08 MB PDF)

Table S2 Species database information for inhibin α-subunit analysis in Fig. 1A and Fig. 1C.
(0.07 MB PDF)

Table S3 Oligonucleotide primers used for deletion mutagenesis.
(0.04 MB PDF)
