# Supplementary material for: Phylogenomic Analyses Reveal the Evolutionary Origin of the Inhibin α-Subunit, a Unique TGFβ Superfamily Antagonist
Source: PLoS One. 2010 Mar 4;5(3):e9457. doi: 10.1371/journal.pone.0009457 (PMC2832003; doi:10.1371/journal.pone.0009457)

Figure S1. Schematic representation of the human and chicken inhibin α and human βA pre-pro subunits. **i,** The entire human pro-αN-αC is depicted. The full length unprocessed subunit is 366 amino acids. The mature domain (αC) is contained in the 3' end of this subunit and is cleaved by the proconvertase enzyme at amino acid 232. The red color shows the human αC domain. The N-terminal extension region of the human α-subunit is shown in green bar, the proline-rich 'wrist region' of the human α-subunit is shown in blue bar. **ii,** The entire chicken pro-αN-αC is depicted. The full length unprocessed subunit is 328 amino acids. The mature domain (αC) is cleaved by the proconvertase enzyme at amino acide 215. The yellow color shows the chicken αC. The N-terminal extension region of the chicken α-subunit is shown in pink bar. **iii,** The entire human pro-β_A is depicted. The full length unprocessed subunit is 426 amino acids. The mature domain (β_A) is contained in the 3' end of this subunit and is cleaved by the proconvertase enzyme at amino acide 310. The black color shows human β_A. The white bar represents the N-terminal region of the mature βA-subunit, with the crosshatch pattern bar indicating the wrist α–helical region. The black bars show the antibody binding sites that are indicated for the R1, PO14, and PO23 inhibin α–subunit-specific monoclonal antibodies, as well as for pAb βA. The proconvertase enzymecleavage sites were presented by black arrow. The 'ψ' symbol indicates glycosylation sites.

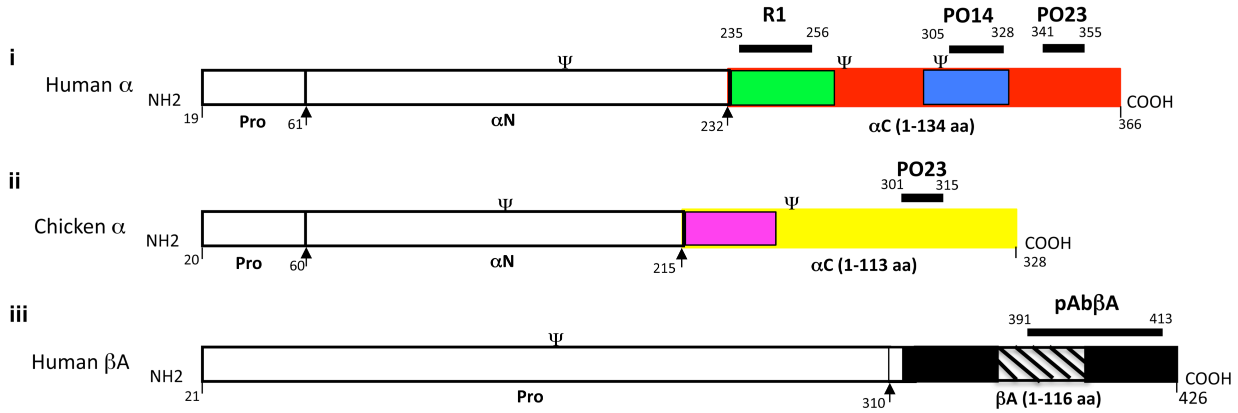

Supplement: Figure S1 — Schematic representation of the human and chicken inhibin α and human βA pre-pro subunits. i, The entire human pro-αN-αC is depicted. The full length unprocessed subunit is 366 amino acids. The mature domain (αC) is contained in the 3′ end of this subunit and is cleaved by the proconvertase enzyme at amino acid 232. The red color shows the human αC domain. The N-terminal extension region of the human α-subunit is shown in green bar, the proline-rich “wrist region” of the human α-subunit is shown in blue bar. ii, The entire chicken pro- αN-αC is depicted. The full length unprocessed subunit is 328 amino acids. The mature domain (αC) is cleaved by the proconvertase enzyme at amino acide 215. The yellow color shows the chicken αC. The N-terminal extension region of the chicken α-subunit is shown in pink bar. iii, The entire human pro-βA is depicted. The full length unprocessed subunit is 426 amino acids. The mature domain (βA) is contained in the 3′ end of this subunit and is cleaved by the proconvertase enzyme at amino acide 310. The black color shows human βA. The white bar represents the N-terminal region of the mature βA-subunit, with the crosshatch pattern bar indicating the wrist α-helical region. The black bars show the antibody binding sites that are indicated for the R1, PO14, and PO23 inhibin α-subunit-specific monoclonal antibodies, as well as for pAb βA. The proconvertase enzymecleavage sites were presented by black arrow. The symbol indicates glycosylation sites. (0.34 MB PDF) [file pone.0009457.s001.pdf]
