# Supplementary material for: Phylogenomic Analyses Reveal the Evolutionary Origin of the Inhibin α-Subunit, a Unique TGFβ Superfamily Antagonist
Source: PLoS One. 2010 Mar 4;5(3):e9457. doi: 10.1371/journal.pone.0009457 (PMC2832003; doi:10.1371/journal.pone.0009457)
Supplement: Figure S2 — Large-scale estimate of inhibin/activin phylogeny. A) Maximum likelihood (ML) estimate of phylogeny for genes retrieved from annotated animal genomes by BLAST (Altschul et al., 2009) searches. The alignment included only the TGFβ domain of genes that have human inhibin/activin genes as their top hit (they all satisfied the bidirectional best hit criterion). This analysis was used the WAG (Whelan and Goldman, 2001) model with the a proportion of sites assumed to be invariant and the remaining sites at different rates drawn from a -distribution (with a shape parameter estimated by ML). Support for clades reflects the percentage of 100 bootstrap replicates. B) Alignment used for part A of this figure, with the 3 amino acid insertion that unites the lancelet (Branciostoma) α-subunit-like protein with the vertebrate (in this alignment, human and zebrafish) inhibin α-subunit highlighted using yellow. C) Alignment for the lancelet α-subunit-like protein with the lancelet β protein mature domain sequence. D) Alignment for the mature domain sequences between the human inhibin α-subunit, chicken inhibin α-subunit and human inhibin βA subunit. The red box that used in the C) and D) highlighted the regions that we focused on. (1.45 MB PDF) [file pone.0009457.s002.pdf]

Figure S2. Large-scale estimate of inhibin/activin phylogeny. A) Maximum likelihood (ML) estimate of phylogeny for genes retrieved from annotated animal genomes by BLAST (Altschul et al., 2009) searches. The alignment included only the TGFβ domain of genes that have human inhibin/activin genes as their top hit (they all satisfied the bidirectional best hit criterion). This analysis was used the WAG (Whelan and Goldman, 2001) model with the a proportion of sites assumed to be invariant and the remaining sites at different rates drawn from a Γ-distribution (with a shape parameter estimated by ML). Support for clades reflects the percentage of 100 bootstrap replicates. **B)** Alignment used for part A of this figure, with the 3 amino acid insertion that unites the lancelet (*Branciostoma*) α-subunit-like protein with the vertebrate (in this alignment, human and zebrafish) inhibin α-subunit highlighted using yellow. **C)** Alignment for the lancelet α-subunit-like protein with the lancelet β protein mature domain sequence. **D)** Alignment for the mature domain sequences between the human inhibin α-subunit, chicken inhibin α-subunit and human inhibin βA subunit. The red box that used in the **C)** and **D)** highlighted the regions that we focused on.

A

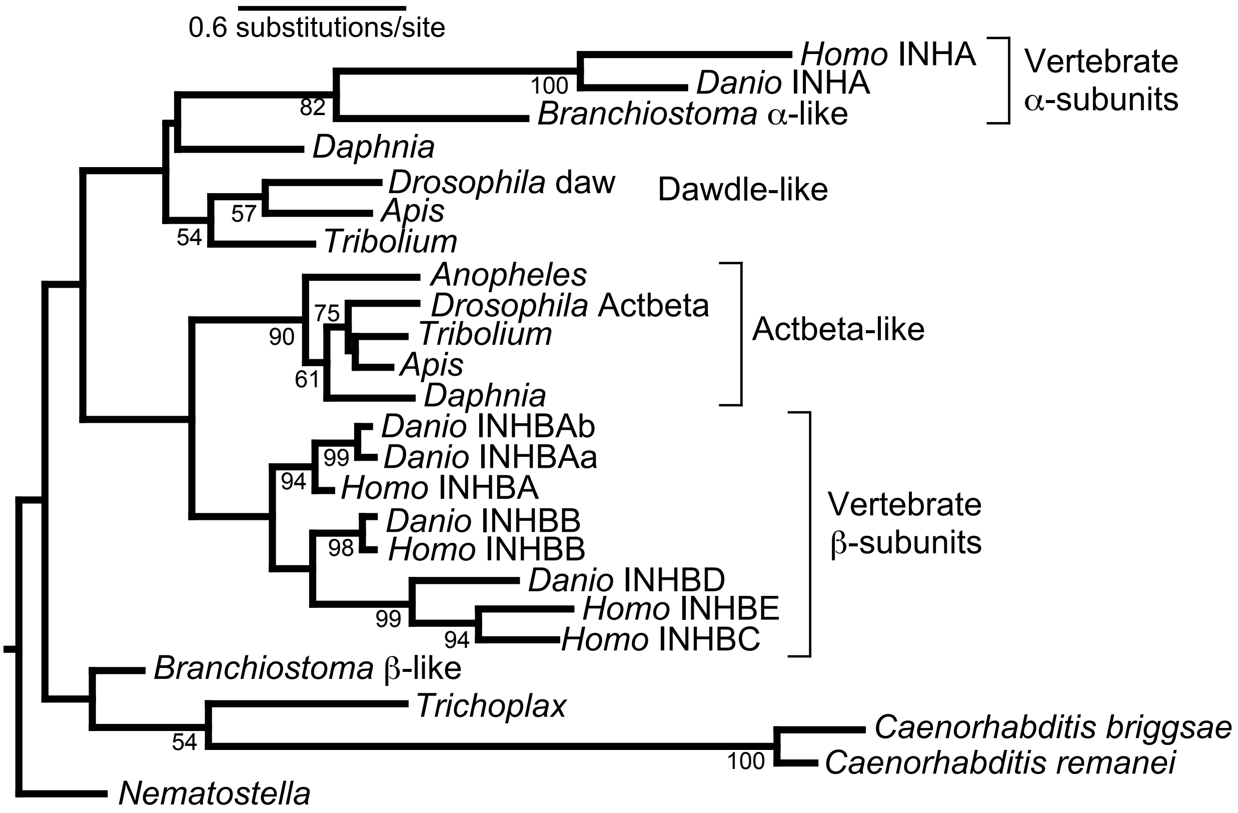

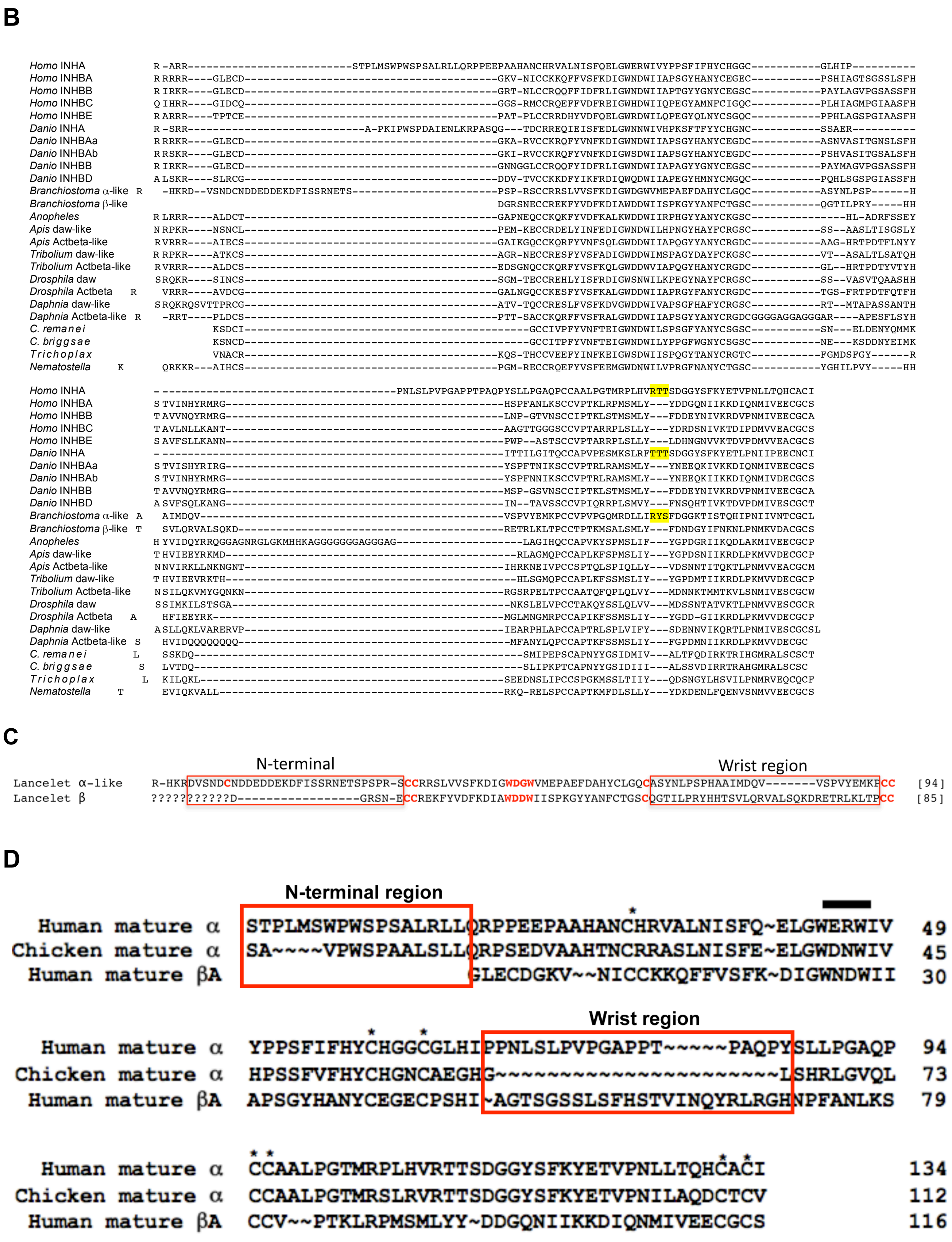
B
Homo INHA
Homo INHBA
Homo INHBB
Homo INHBC
Homo INHBE
Danio INHA
Danio INHBAa
Danio INHBAb
Danio INHBB
Danio INHBD
Branchiostoma α-like
Branchiostoma β-like
Anopheles
Apis daw-like
Apis Actbeta-like
Tribolium daw-like
Tribolium Actbeta-like
Drosphila daw
Drosphila Actbeta
Daphnia daw-like
Daphnia Actbeta-like
C. remanei
C. briggsae
Trichoplax
Nematostella
C
N-terminal
Wrist region
Lancelet α-like
Lancelet β
[94]
[85]
D
N-terminal region
Wrist region
Human mature α
Chicken mature α
Human mature βA
49
45
30
94
73
79
134
112
116

References:

Altschul, S.F., Gertz, E.M., Agarwala, R., Schäffer, A.A., and Yu, Y.K. (2009). PSI-BLAST pseudocounts and the minimum description length principle. Nucleic Acids Res *37*, 815-824.

Altschul, S.F., Madden, T.L., Schäffer, A.A., Zhang, J., Zhang, Z., Miller, W., and Lipman, D.J. (1997). Gapped BLAST and PSI-BLAST: a new generation of protein database search programs. Nucleic Acids Res *25*, 3389-3402.

Pigliucci, M. (2007). Do we need an extended evolutionary synthesis? Evolution *61*, 2743-2749.

Whelan, S., and Goldman, N. (2001). A general empirical model of protein evolution derived from multiple protein families using a maximum-likelihood approach. Mol Biol Evol *18*, 691-699.
