# Supplementary material for: Phylogenomic Analyses Reveal the Evolutionary Origin of the Inhibin α-Subunit, a Unique TGFβ Superfamily Antagonist
Source: PLoS One. 2010 Mar 4;5(3):e9457. doi: 10.1371/journal.pone.0009457 (PMC2832003; doi:10.1371/journal.pone.0009457)

Fig S3. Detailed version of the inhibin/activin phylogeny presented in Fig. 1C. The ML analysis used the JTT+Γ+invariant sites model, as described in the text. Support for specific groups reflects the percentage of 100 bootstrap replicates (only values ≥50%). The version in the text indicates the occurance of indel mutations, including the indels shown to have a major functional impact.

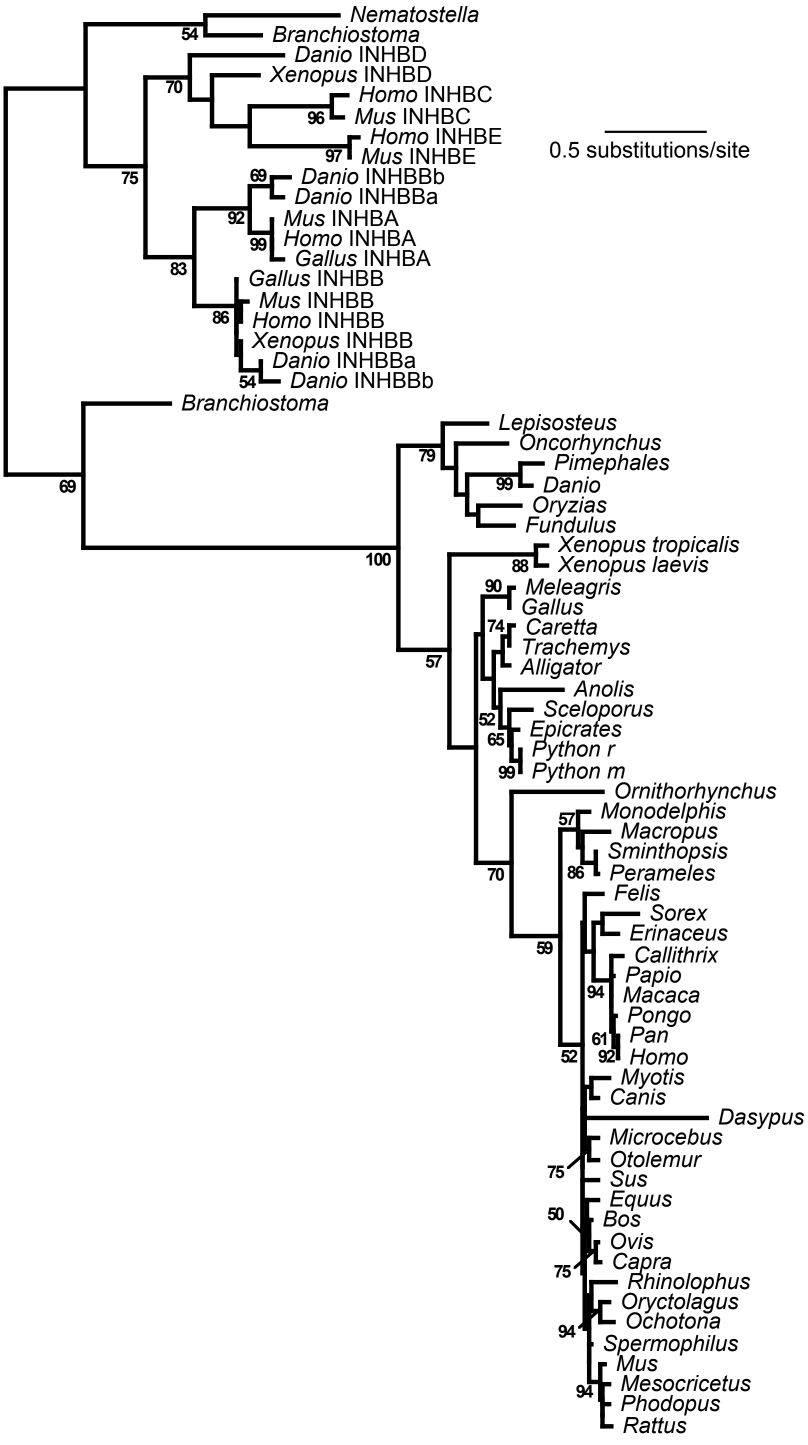

Supplement: Figure S3 — Detailed version of the inhibin/activin phylogeny presented in Fig. 1C. The ML analysis used the JTT+ ζ+invariant sites model, as described in the text. Support for specific groups reflects the percentage of 100 bootstrap replicates (only values ≥50%). The version in the text indicates the occurence of indel mutations, including the indels shown to have a major functional impact. (0.08 MB PDF) [file pone.0009457.s003.pdf]
