# Supplementary material for: Phylogenomic Analyses Reveal the Evolutionary Origin of the Inhibin α-Subunit, a Unique TGFβ Superfamily Antagonist
Source: PLoS One. 2010 Mar 4;5(3):e9457. doi: 10.1371/journal.pone.0009457 (PMC2832003; doi:10.1371/journal.pone.0009457)
Supplement: Figure S4 — Detail of mutations within human inhibin αC and βA-subunits. A) The schematic representation of wild-type human αC and human βA-subunits. The letters “A”, “B”. “C”, “D”, “E” and “F” denote the six candidate regions targeted for deletion. B) The residue numbers and sequences for the six regions targeted for deletion and used for generating the inhibin α-subunit deletion mutants and inhibin βA-subunit chimera mutants. C) Design and naming conventions for the α-subunit and β-subunit mutants. (0.32 MB PDF) [file pone.0009457.s004.pdf]

Figure S4. Detail of mutations within human inhibin αC and βA-subunits. A) The schematic representation of wild-type human αC and human βA-subunits. The letters "A," "B," "C," "D," "E" and "F" denote the six candidate regions targeted for deletion. **B)** The residue numbers and sequences for the six regions targeted for deletion and used for generating the inhibin α-subunit deletion mutants and inhibin β_A-subunit chimera mutants. **C)** Design and naming conventions for the α-subunit and β_A-subunit mutants.

A

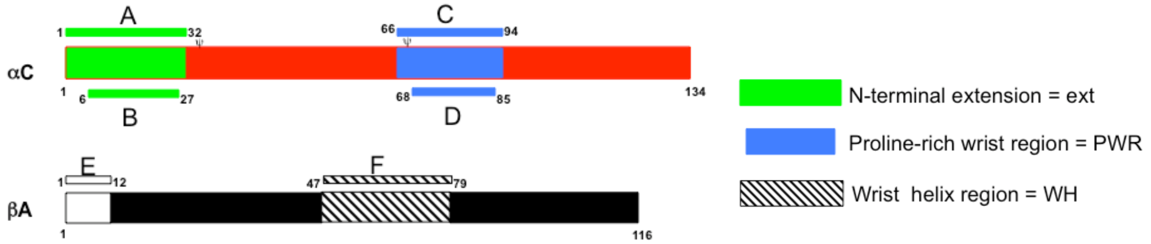

B

Region		Amino acid location	Sequence
A	α	1-32	STPLMSWPWSPSALRLLQRPPEEPAAHANCHR
B		6-27	SWPWSPSALRLLQRPPEEPAAH
C		66-94	HIPPNLSLPVPGAPPTPAQPYSLLPGAQP
D		68-85	PPNLSLPVPGAPPTPAQP
E	βA	1-12	GLECDGKVNICC
F		47-79	HIAGTSGSSLSFHSTVINQYRLRGHNPFANLKS

C

Wild type inhibinA	mutants	mutants detail
α^{Hwt}/βA	α^{Hext-}/βA	Remove region "B",heterodimer with βA
α^{Chwt}/βA	α^{Hwr-}/βA	Remove region "D",heterodimer with βA
βA /βA	$\alpha^{Hext-wr-}$/βA	Remove region "B" and "D",heterodimer with βA
	$βA^{Hext+}$/ $βA^{Hext+}$	Replace region "E" with region "A"
	$βA^{hwr+}$/ $βA^{hwr+}$	Replace region "F" with region "C"
	$βA^{HD}$/ $βA^{HD}$	Remove region "F"

Note: H=human, Ch=chicken, wt=wildtype, ext=extension region, wr=wrist region, HD=Helix deletion
