# Supplementary material for: Phylogenomic Analyses Reveal the Evolutionary Origin of the Inhibin α-Subunit, a Unique TGFβ Superfamily Antagonist
Source: PLoS One. 2010 Mar 4;5(3):e9457. doi: 10.1371/journal.pone.0009457 (PMC2832003; doi:10.1371/journal.pone.0009457)
Supplement: Figure S5 — Negative control for the Fig. 3B and Tandem MS-MS fragment mass mapping. A) Left, immunoblot of media from cells expressing wild type human α-subunit alone and human inhibin A under non-reducing or reducing conditions. Middle, immunoblot of media from cells expressing human free α or human inhibin A that were labeled with [35S]-cysteine for 48 hours. Media were collected and immunoprecipitated with an anti-α-subunit monoclonal antibody PO23 and then subjected to SDS-PAGE under non-reducing conditions and autoradiography. Right, negative control immunoblot of mouse IgG-immunoprecipitated [35S]-cysteine labeled media. Labels 1, 2, 3 and 4 indicate media from cells expressing αHwt/αHwt, αHwt/βA, αChwt/αChwt and αChwt, respectively. B) The culture media from the chicken α-subunit alone expression cell was collected and immunoprecipitated with an anti-α-subunit monoclonal antibody PO23 and subjected to SDS-PAGE under non-reducing conditions. The gel was stained with Coomasie. The band marked with “*” is supposed chicken α-α homodimer band The molecular weight is around 30 kDa. C) The band was digested with MS grade trypsin and subjected to tandem MS- MS analysis. Using the SwissProt database, the resulting fragment from the band mapped to the chicken inhibin α-subunit mature domain. The matched fragment is highlighted in red. (3.97 MB PDF) [file pone.0009457.s005.pdf]

Figure S5. Negative control for the Fig 3B and Tandem MS-MS fragment mass mapping. **A)** Left, immunoblot of media from cells expressing wild type human α-subunit alone and human inhibin A under non-reducing or reducing conditions. Middle, immunoblot of media from cells expressing human free α or human inhibin A that were labeled with $[^{35}S]$-cysteine for 48 hours. Media were collected and immunoprecipitated with an anti-α-subunit monoclonal antibody PO23 and then subjected to SDS-PAGE under non-reducing conditions and autoradiography. Right, negative control immunoblot of mouse IgG-immunoprecipitated $[^{35}S]$-cysteine labeled media. Labels 1, 2, 3 and 4 indicate media from cells expressing $\alpha^{Hwt}/\alpha^{Hwt}$, $\alpha^{Hwt}/\beta A$, $\alpha^{Chwt}/\alpha^{Chwt}$ and α^{Chwt} , respectively. **B)** The culture media from the chicken α-subunit alone expression cell was collected and immunoprecipitated with an anti-α-subunit monoclonal antibody PO23 and subjected to SDS-PAGE under non-reducing conditions. The gel was stained with Coomasie. The band marked with "*" is supposed chicken α-α homodimer band The molecular weight is around 30kDa. **C)** The band was digested with MS grade trypsin and subjected to tandem MS-MS analysis. Using the SwissProt database, the resulting fragment from the band mapped to the chicken inhibin α-subunit mature domain. The matched fragment is highlighted in red.

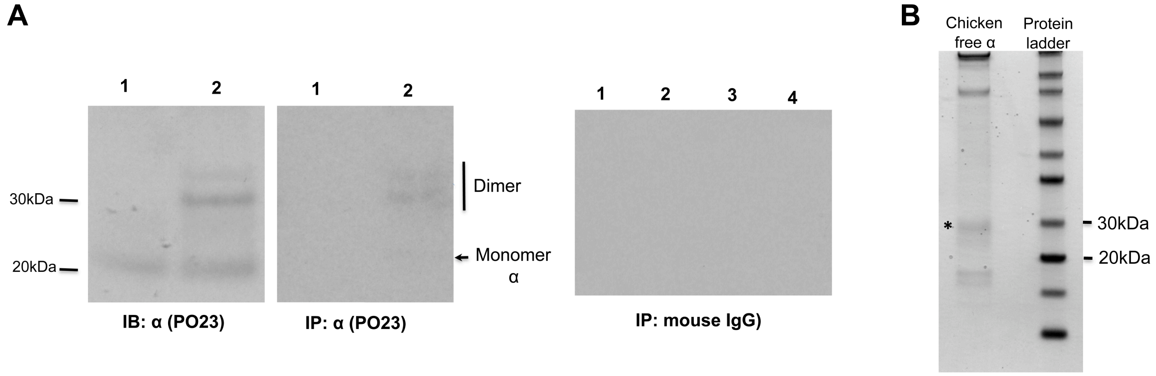

C

20. gi|71895193 Mass: 35830 Score: 62 Queries matched: 3
inhibin alpha subunit [Gallus gallus]

Query	Observed	Mr(expt)	Mr(calc)	Delta	Miss	Score	Expect	Rank	Peptide
196	1062.3755	1061.3682	1061.4666	-0.0983	0	40	5.4	1	R.TTSDGGYSFK.Y
197	1062.4878	1061.4805	1061.4666	0.0140	0	(29)	80	1	R.TTSDGGYSFK.Y

Chicken inhibin a full length sequence:

MLLLLHLLPAVLPASALGSCTGAGADRQLVLAKVRARVLEHLSPPAMQEPQKDVRR
VHRRDVLEEVEVPPEEQEDTSQVILFPSTDVPCEPTQPDKLLEEEGIFTYLFQPSA
HALSRTLTSAQLWFYSGPSAAPNHSAPAVLTLSPQGRVPVVATASRTPEHWTVFDF
GPDALPQLAQPLFVLLVRCPGCPCLADGDKMPFLVATTRAKAAGRARRSAVPWSPA
ALSLLQRPSEDVAAHTNCRRASLNISFEELGWDNWIVHPSSFVFHYCHGNCAEGHG
LSHRLGVQLCCAALPGTMRSLRVRTTSDGGYSFKYETVPNILAQDCTCV
