# Supplementary material for: Phylogenomic Analyses Reveal the Evolutionary Origin of the Inhibin α-Subunit, a Unique TGFβ Superfamily Antagonist
Source: PLoS One. 2010 Mar 4;5(3):e9457. doi: 10.1371/journal.pone.0009457 (PMC2832003; doi:10.1371/journal.pone.0009457)

Figure S6. Immunoblots of media from cells expressing wild type human and chicken inhibin A and various human α-subunit deletion mutants run under reducing conditions. Labels 1', 2', 3', 4' and 5' indicate media from cells expressing α^{Hwt}/βA, α^{Chwt}/βA, α^{Hext-}/βA, α^{HPWR-}/βA and $\alpha^{Hext-PWR-}$/βA, respectively. **A)** Blot was detected by an anti-α-subunit monoclonal antibody PO23. **B)** Blot was detected by anti-βA-subunit polyclonal antibody.

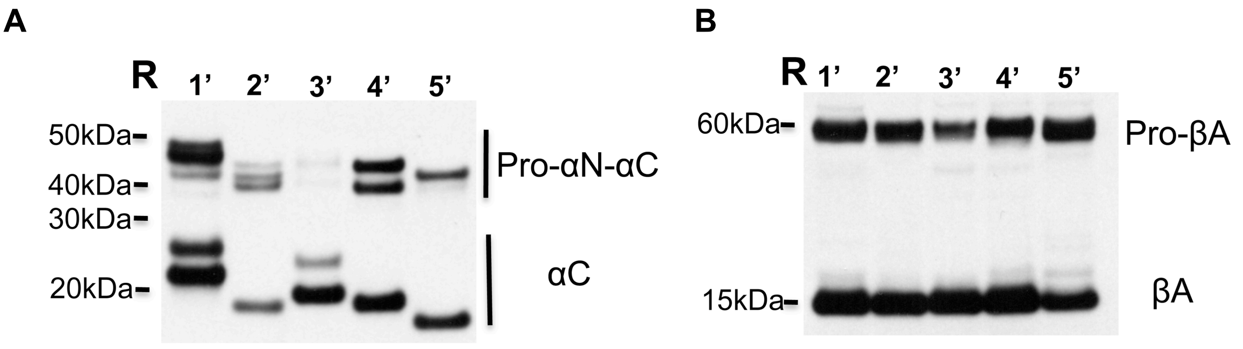

Supplement: Figure S6 — Immunoblots of media from cells expressing wild type human and chicken inhibin A and various human α-subunit deletion mutants run under reducing conditions. Labels 1′, 2′, 3′, 4′ and 5′ indicate media from cells expressing αHwt/βA, αChwt/βA, αHext-/βA, αHPWR-/βA and αHext-PWR-/βA, respectively. A) Blot was detected by an anti-α-subunit monoclonal antibody PO23. B) Blot was detected by anti-βA-subunit polyclonal antibody. (1.58 MB PDF) [file pone.0009457.s006.pdf]
