# Supplementary material for: Phylogenomic Analyses Reveal the Evolutionary Origin of the Inhibin α-Subunit, a Unique TGFβ Superfamily Antagonist
Source: PLoS One. 2010 Mar 4;5(3):e9457. doi: 10.1371/journal.pone.0009457 (PMC2832003; doi:10.1371/journal.pone.0009457)

Figure S7. Standard curves for biotinylated activin A/inhibin A binding to ActRIIB. A) Standard curve for biotinylated activin A; EC_{50}=1.10 nM. Related to **Fig 4A. B)** Standard curve for biotinylated inhibin A; EC_{50}=2.73 nM. Related to **Fig 4C.**

A **B**

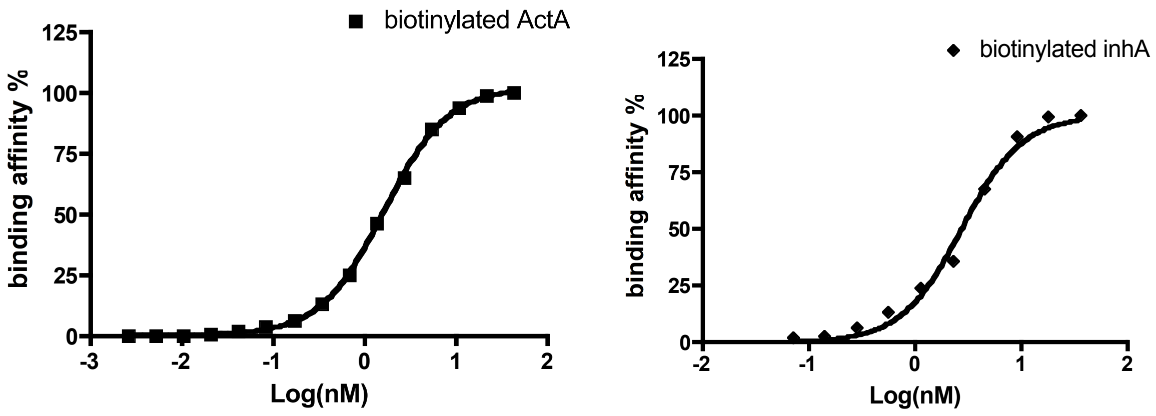

Supplement: Figure S7 — Standard curves for biotinylated activin A/inhibin A binding to ActRIIB. A) Standard curve for biotinylated activin A; EC50 = 1.10 nM. Related to Fig. 4A. B) Standard curve for biotinylated inhibin A; EC = 2.73 nM. Related to Fig. 4C. (0.12 MB PDF) [file pone.0009457.s007.pdf]
