# Supplementary material for: Phylogenomic Analyses Reveal the Evolutionary Origin of the Inhibin α-Subunit, a Unique TGFβ Superfamily Antagonist
Source: PLoS One. 2010 Mar 4;5(3):e9457. doi: 10.1371/journal.pone.0009457 (PMC2832003; doi:10.1371/journal.pone.0009457)

Figure S8. Negative and positive controls for the co-immunoprecipitation experiments presented in Fig. 4B. COS7 cells were transiently transfect with ActRIIB-HA with either pCDNA3 or betaglycan-c-myc (BG-c-myc). **A)** Cells were treated with 100 ng/ml human activin A or empty vector culture media (EV media). The cell lysate was immunoprecipitated by monoclonal anti-HA or mouse IgG, followed by immunoblot with anti-β_A-subunit polyclonal antibody. **B)** Cells were treated with 100 ng/ml human activin A (panel 1); empty vector (panel 2), human wild type inhibin A (panel 3); chicken wild type inhibin A (panel 4); or culture media from cells expressing human inhibin A deletion mutants $\alpha^{Hext-}/\beta A$ (panel 5), $\alpha^{HPWR-}/\beta A$ (panel 6), or $\alpha^{Hext-PWR-}/\beta A$ (panel 7). Proteins were detected in the cell lysates by immunoblotting with either monoclonal anti-c-myc or monoclonal anti-HA antibodies.

A

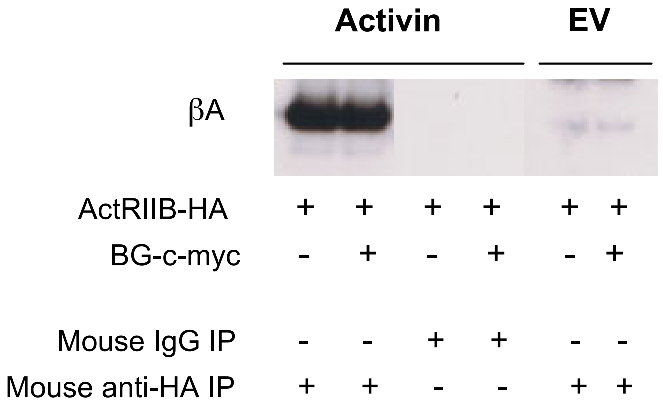

B

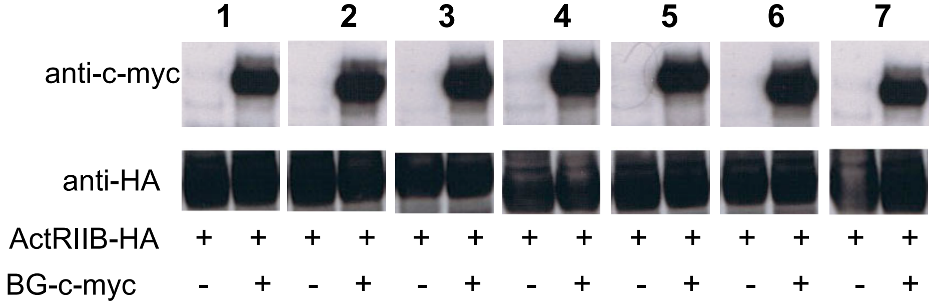

Supplement: Figure S8 — Negative and positive controls for the co-immunoprecipitation experiments presented in Fig. 4B. COS7 cells were transiently transfected with ActRIIB-HA with either pCDNA3 or betaglycan-c-myc (BG-c-myc). A) Cells were treated with 100 ng/ml human activin A or empty vector culture media (EV media). The cell lysate was immunoprecipitated by monoclonal anti-HA or mouse IgG, followed by immunoblot with anti-βA-subunit polyclonal antibody. B) Cells were treated with 100 ng/ml human activin A (panel 1); empty vector (panel 2), human wild type inhibin A (panel 3); chicken wild type inhibin A (panel 4); or culture media from cells expressing human inhibin A deletion mutants αHext-/βA (panel 5), αHPWR-/βA (panel 6), or αHext-PWR-/βA (panel 7). Proteins were detected in the cell lysates by immunoblotting with either monoclonal anti-c-myc or monoclonal anti-HA antibodies. (1.56 MB PDF) [file pone.0009457.s008.pdf]
