# Supplementary material for: Phylogenomic Analyses Reveal the Evolutionary Origin of the Inhibin α-Subunit, a Unique TGFβ Superfamily Antagonist
Source: PLoS One. 2010 Mar 4;5(3):e9457. doi: 10.1371/journal.pone.0009457 (PMC2832003; doi:10.1371/journal.pone.0009457)
Supplement: Table S1 — Species database information for TGFβ family members analyzed in Fig. 1A and Fig. 1B. (0.08 MB PDF) [file pone.0009457.s009.pdf]

Table S1. Species database information for TGFβ family members analyzed in Fig 1A and Fig 1B.

Vertebrate

		Danio rerio	*Xenopus laevis*	*Gallus gallus*	*Mus musculus*	*Homo sapiens*
AMH					NP_031471	NP_000470
GDF1					NP_032133	NP_001483
GDF3					NP_032134	NP_065685
GDF11					NP_034402	NP_005802
Inhibin-alpha		NP_001038669	NP_001106341/ NP_001106349	NP_001026428	NP_034694	NP_002182
Inhibin-beta	A	NP_001018166/ 56790252	-	NP_990727	NP_032406	NP_002183
	B	NP_571143	NP_001084055	NP_990537	NP_032407	NP_002184
	C	-	-	-	NP_034695	NP_005529
	D	XP_700865	NP_001079333	-	-	-
	E	-	-	-	NP_032408	NP_113667
Lefty					NP_034224/ NP_796073	NP_003231/ NP_066277
Myostatin					NP_034964	NP_005250
Nodal					NP_038639	NP_060525
TGF-beta	1				NP_035707	NP_000651
	2				NP_033393	NP_003229/ NP_001129071
	3				NP_033394	NP_003230

Insects

	Hymenoptera		Coleoptera	Diptera		
	Nasonia vitripennis	*Apis mellifera*	*Tribolium castaneum*	*Anopheles gambiae*	*Aedes aegypti*	*Culex quinquefasciatus*
Activin	XP_001602284	XP_001123044	XP_966908			
Activin-like protein		XP_001122210	XP_970355			
Maverick	XP_001606148	XP_001122118		XP_320452	XP_001656215	XP_001870685
Myoglianin						
Myostatin	XP_001602255		XP_966819	XP_309077	XP_001656630	XP_001842764

Drosophila

	ananassae	***erecta***	***grimshawi***	***melanogaster***	***mojavensis***	***persimilis***
Activin	XP_001967557	XP_001982690	XP_001996489	NP_651942	XP_002011430	-
Activin-like protein	XP_001961464	XP_001968468	XP_001993123	NP_523461	XP_002002273	XP_002028363
Maverick	XP_001953456	XP_001982717	-	NP_524626/ NP_001014690	XP_002011465	XP_002027069
Myoglianin	XP_001966254	XP_001982711	XP_001996544	NP_524627	XP_002011400	XP_002027072

	pseudoobscura	***sechellia***	***simulans***	***virilis***	***willistoni***	***yakuba***
Activin	XP_001352350	XP_002044322	-	XP_002059698	XP_002072630	XP_002099638
Activin-like protein	XP_002133178	XP_002037633	XP_002077940	XP_002057795	XP_002066964	XP_002087729
Maverick	XP_001352269	XP_002043687	-	XP_002059723	-	XP_002099611
Myoglianin	XP_001352370	XP_002043694	-	XP_002059743	XP_002072620	XP_002099617
