# Supplementary material for: Phylogenomic Analyses Reveal the Evolutionary Origin of the Inhibin α-Subunit, a Unique TGFβ Superfamily Antagonist
Source: PLoS One. 2010 Mar 4;5(3):e9457. doi: 10.1371/journal.pone.0009457 (PMC2832003; doi:10.1371/journal.pone.0009457)
Supplement: Table S2 — Species database information for inhibin α-subunit analysis in Fig. 1A and Fig. 1C. (0.07 MB PDF) [file pone.0009457.s010.pdf]

Table S2. Species database information for inhibin α-subunit analysis in Fig 1A and Fig 1C.

Classification			Name	Genbank or Ensembl ID
Actinopterygii	Semionotiformes		*Lepisosteus platyrhinchus* (Florida gar)	FJ457910
	Teleostei	Euteleostei	*Oncorhynchus mykiss* (rainbow trout)	NP_001117672
			Fundulus heteroclitus (killifish)	AAW02847
			Oryzias latipes (Japanese medaka)	ENSORLP00000002713
		Otocephala	*Danio rerio* (zebrafish)	NP_001038669
			Pimephales promelas	ABS30832
Amphibia	Anura		*Xenopus laevis* (African clawed frog)	NP_001106349
			Xenopus (Silurana) *tropicalis* (western clawed frog)	NP_001027522
Sauropsida	Archosauria	Crocodylidae	*Alligator mississippiensis* (American alligator)	FJ457911
		Aves	*Gallus gallus* (chicken)	NP_001026428
			Meleagris gallopavo (turkey)	AAK21264
	Testudines		*Caretta caretta* (Loggerhead turtle)	FJ457912
			Trachemys scripta (red-eared slider turtle)	FJ457913
	Squamata		*Anolis carolinensis* (green anole)	120:3014921:3015701:1
			Sceloporus jarrovi	FJ457914
			Epicrates cenchria (Rainbow Boa)	FJ457915
			Python molurus bivittatus (Burmese Python)	FJ457916
			Python regius (Ball pyson)	FJ457917
Mammalia	Monotremata		*Ornithorhynchus anatinus* (platypus)	ENSOANP00000028722
	Metatheria		*Monodelphis domestica* (gray short-tailed opossum)	XP_001362298
			Perameles gunnii (eastern barred bandicoot)	FJ457918
			Sminthopsis crassicaudata (fat-tailed dunnart)	FJ457919
			Macropus eugenii (tammar wallaby)	FJ457920
	Eutheria	Afrotheria	*Echinops telfairi* (small Madagascar hedgehog)	ENSETEP00000007737
		Xenarthra	*Dasypus novemcinctus* (nine-banded armadillo)	ENSDNOP00000003040
		Insectivora	*Erinaceus europaeus* (western European hedgehog)	ENSEEUP00000012588
			Sorex araneus (European shrew)	ACE77661
		Chiroptera	*Myotis lucifugus* (little brown bat)	ENSMLUP00000009480
			Rhinolophus ferrumequinum (greater horseshoe bat)	ACC64569
		Carnivora	*Canis lupus familiaris* (dog)	XP_545660
			Felis catus (domestic cat)	AAP83317
		Perissodactyla	*Equus caballus* (horse)	NP_001075379
		Cetartiodactyla	*Sus scrofa* (Pigliucci)	NP_999354
			Bos taurus (cattle)	P07994
			Capra hircus (goat)	ABR13682
			Ovis aries (sheep)	P38440
		Primates	*Otolemur garnettii* (small-eared galago)	ENSOGAP00000000591
			Microcebus murinus (gray mouse lemur)	ENSMICP00000007418
			Callithrix jacchus (white-tufted-ear marmoset)	ABY89812
			Macaca mulatta (rhesus monkey)	NP_001028127
			Papio anubis (olive baboon)	ABY67214
			Pongo pygmaeus (Bornean orangutan)	ENSPPYP00000014765
			Homo sapiens (human)	NP_002182
			Pan troglodytes (chimpanzee)	XP_001148064
		Lagomorpha	*Ochotona princeps* (American pika)	ENSOPRP00000009166
			Oryctolagus cuniculus (rabbit)	ENSOCUP00000009768
		Rodentia	*Spermophilus tridecemlineatus* (thirteen-lined ground squirrel)	ENSSTOP00000011140
			Mesocricetus auratus (golden hamster)	BAD95496
			Phodopus sungorus (Siberian hamsters)	AAL67332
			Mus musculus (house mouse)	NP_034694
			Rattus norvegicus (Norway rat)	NP_036722
