# Supplementary material for: Phylogenomic Analyses Reveal the Evolutionary Origin of the Inhibin α-Subunit, a Unique TGFβ Superfamily Antagonist
Source: PLoS One. 2010 Mar 4;5(3):e9457. doi: 10.1371/journal.pone.0009457 (PMC2832003; doi:10.1371/journal.pone.0009457)
Supplement: Table S3 — Oligonucleotide primers used for deletion mutagenesis. (0.04 MB PDF) [file pone.0009457.s011.pdf]

Table S3. Oligonucleotide primers used for deletion mutagenesis.

Primer	Sequence
α^{Hext-} forward	TCAACTCCCCTGATGGCCAACTGCCACAGAGTAGC
α^{Hext-} reverse	GCTACTCTGTGGCAGTTGGCCATCAGGGGAGTTGA
α^{HPWR-} forward	TGTGGGCTGCACATCTACTCCTTGCTGCCAGGGGC
α^{HPWR-} reverse	GCCCCTGGCAGCAAGGAGTAGATGTGCAGCCCACAG
βA^{WHD} forward	GAGGGTGAGTGCCCGAGCTGCTGTGTGCCCACCAAG
βA^{WHD} reverse	CTTGGTGGGCACACAGCAGCTCGGGCACTCACCCTC
